# Efficient Production of Adipic Acid by a Two‐Step Catalytic Reaction of Biomass‐Derived 2,5‐Furandicarboxylic Acid

**DOI:** 10.1002/cssc.202200375

**Published:** 2022-04-01

**Authors:** Anh Vy Tran, Seok‐Kyu Park, Hye Jin Lee, Tae Yong Kim, Younhwa Kim, Young‐Woong Suh, Kwan‐Young Lee, Yong Jin Kim, Jayeon Baek

**Affiliations:** ^1^ Green and Sustainable Materials R&D Department Korea Institute of Industrial Technology (KITECH) 89 Yangdeagiro-gil Ipjang-myeon Cheonan-si 31056 Republic of Korea; ^2^ Department of Chemical and Biological Engineering Korea University 145 Anam-ro, Seongbuk-gu Seoul 02841 Republic of Korea; ^3^ Department of Chemical Engineering Pohang University of Science and Technology 77 Cheongam-ro, Nam-gu Pohang Gyeongbuk 37673 Republic of Korea; ^4^ School of Chemical and Biological Engineering Seoul National University Gwanak-ro, Gwanak-gu Seoul 08826 Republic of Korea; ^5^ Department of Chemical Engineering Hanyang University Seoul 04763 Republic of Korea; ^6^ Department of Green Process and System Engineering University of Science and Technology (UST) 217 Gajeong-ro, Yuseong-gu Daejeon-si 34113 Republic of Korea

**Keywords:** biomass valorization, carboxylic acids, heterogeneous catalysis, hydrodeoxygenation, ruthenium

## Abstract

Efficient catalytic ring‐opening coupled with hydrogenation is a promising but challenging reaction for producing adipic acid (AA) from 2,5‐furan dicarboxylic acid (FDCA). In this study, AA synthesis is carried out in two steps from FDCA via tetrahydrofuran‐2,5‐dicarboxylic acid (THFDCA) over a recyclable Ru/Al_2_O_3_ and an ionic liquid, [MIM(CH_2_)_4_SO_3_H]I (MIM=methylimidazolium) to deliver 99 % overall yield of AA. Ru/Al_2_O_3_ is found to be an efficient catalyst for hydrogenation and hydrogenolysis of FDCA to deliver THFDCA and 2‐hydroxyadipic acid (HAA), respectively, where ruthenium is more economically viable than well‐known palladium or rhodium hydrogenation catalysts. H_2_ chemisorption shows that the alumina phase strongly affects the interaction between Ru nanoparticles (NPs) and supports, resulting in materials with high dispersion and small size of Ru NPs, which in turn are responsible for the high conversion of FDCA. An ionic liquid system, [MIM(CH_2_)_4_SO_3_H]I is applied to the hydrogenolysis of THFDCA for AA production. The [MIM(CH_2_)_4_SO_3_H]I exhibits superior activity, enables simple product isolation with high purity, and reduces the severe corrosion problems caused by the conventional hydroiodic acid catalytic system.

## Graphical abstract

**AA, my my**: The production of adipic acid (AA) by a two‐step pathway is studied. Ru/Al_2_O_3_ catalyst is used to obtain THFDCA and HAA (see graphic) by hydrogenation of furandicarboxylic acid. The ionic liquid [MIM(CH_2_)_4_SO_3_H]I serves as both a reaction medium for conveying hydrogen and a highly catalytic system for furan ring‐opening toward AA with 99 % yield.

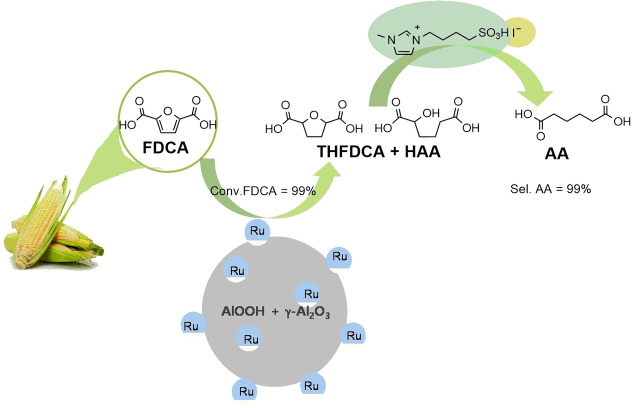

## Introduction

Adipic acid (AA), also known as hexanedioic acid, is widely used as an essential monomer in the manufacturing of Nylon‐66 and is also applied in the production of polyurethane, resins, plasticizers, food, and pharmaceutical additives.^[1]^ The conventional production of AA relies on the hazardous petrochemical‐derived benzene via several steps starting with the hydrogenation of benzene to cyclohexane, followed by the oxidation of cyclohexane to KA oil (ketone‐alcohol oil) over Co^II^ naphthenate catalyst, and further oxidation of KA oil under harsh conditions using a concentrated nitric acid to yield AA.[^1a^, ^1d^, ^2^] This process not only suffers from the high energy consumption but also has drawbacks of using corrosive nitric acid and the generation of undesired N_2_O into the environment. Many studies are currently devoted to developing a green and efficient catalytic conversion route to produce AA from biomass‐derived chemicals, substituting petroleum‐based precursors.[^1d^, ^1e^, ^3^] Among various biomass feedstocks, 5‐hydroxymethylfurfural (HMF) is considered as a versatile platform for producing a variety of value‐added chemicals.[^1a^, ^3b^, ^4^] Several routes have been developed for preparing AA via HMF feedstock. An attractive pathway via 2,5‐furandicarboxylic acid (FDCA) and tetrahydrofuran‐2,5‐dicarboxylic acid (THFDCA) shows that HMF has great potential as an alternative to benzene for the production of AA. The synthetic pathway for AA includes the catalytic oxidation of HMF and subsequent deoxygenation of the product FDCA to AA. Much of the work has been done on the oxidation of HMF toward FDCA, and good results have been reported by using Au, Pt, Pd, and Ru‐based catalysts.^[5]^ However, not many studies have been mentioned on AA production from FDCA. The direct route from FDCA to AA appears attractive since it is a one‐pot pathway. However, it proceeds at high temperatures and pressure of H_2_ over the noble metals as catalysts. For example, Asano et al. reported that a solid Pt‐MoO_*x*_/TiO_2_ converted FDCA into AA with a yield of 21 % at 473 K.^[6]^ Recently, Wei et al. studied that Pt/Nb_2_O_5_ ⋅ *x*H_2_O shows higher activity in AA synthesis due to the Lewis acid sites on Nb_2_O_5_ ⋅ *x*H_2_O surfaces, and it favors the adsorption and activation of the C−O−C bond in the furan ring.^[1a]^ Despite its evident advantages, the direct synthesis of FDCA is plagued by the not yet satisfactory selectivity of AA, and it remains challenging.

Boussie et al. firstly reported the production of AA through sequential hydrogenation of the furan ring to THFDCA over Pd and Rh catalysts, then subsequent conversion of THFDCA into AA using hydroiodic acid (HI).^[7]^ The catalytic hydrogenation of FDCA was reported with Pd/silica and Rh/silica catalysts in acetic acid as solvent. THFDCA yielded up to 88 % after 3 h of reaction at 140 °C and 50 bar of H_2_. Besides, Moore J.A. et al. also reported the hydrogenation of FDCA over a 5 wt % Rh/C with 83 % yield of THFDCA.^[8]^ The subsequent conversion of THFDCA was conducted with hydrobromic acid (HBr) or HI in acetic acid, and a 99 % yield of AA was obtained at 160 °C.^[9]^ Recently, Gilkey et al. described the reaction mechanism and kinetic measurements of the HI‐mediated ring‐opening for producing AA.^[10]^ Many attempts have been made to solve corrosive acidic catalysts, such as using Nafion proton‐exchanged resins or zeolites.[^4a^, ^11^] All these systems have exhibited good catalytic performance for the production of AA with a yield of over 80 % and have helped reduce the corrosion problems. However, the degradability of these catalysts after reaction and the formation of by‐products from the esterification between 2‐hydroxyadipic acid (HAA) and propionic acid remain a hurdle. It is obvious that the environmental effect and economic feasibility of this process have yet to be developed.

**Scheme 1:**
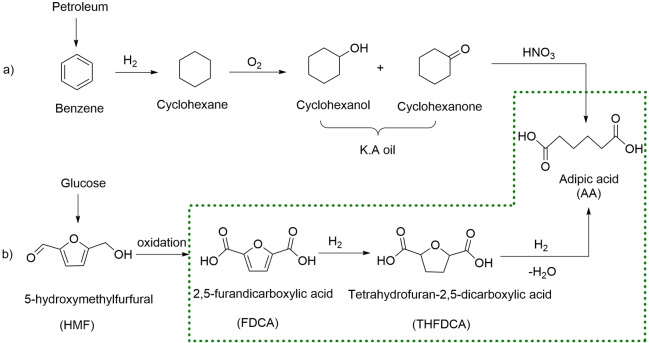
a) Conventional process and b) biomass‐derived chemicals process for the formation of AA (dashed outline represents this study).

Herein, we present the production of AA by a two‐step pathway (Scheme 1). FDCA is converted into THFDCA and HAA over a Ru/Al_2_O_3_ catalyst, which is considered much cheaper than Pd and Rh catalysts. The second step, an iodide‐containing ionic liquid [MIM(CH_2_)_4_SO_3_H]I (MIM=methylimidazolium), is used for the THFDCA ring‐opening and hydrogenation of HAA to produce AA without an organic acid. [MIM(CH_2_)_4_SO_3_H]I system provides efficient catalysis as well as recyclable reaction medium for simple isolation of the desired product.

## Results and Discussion

**Figure 1:**
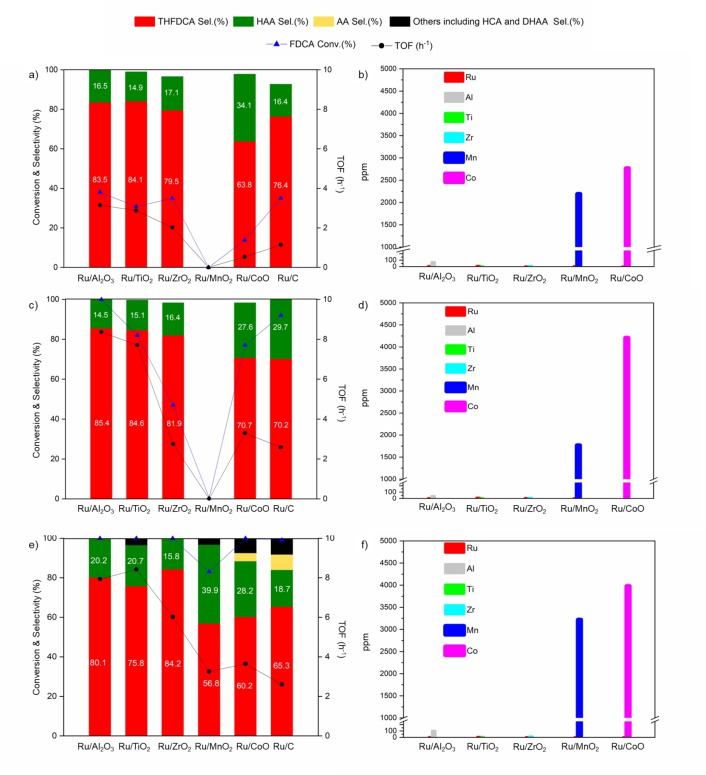
The conversion of FDCA into THFDCA over different kinds of Ru/metal oxide catalysts as a function of reaction temperatures of a) 30 °C, c) 50 °C, and e) 80 °C. ICP analysis of supernatant of different Ru/metal oxides after centrifugation of the reaction solutions at b) 30 °C, d) 50 °C, and f) 80 °C. Conditions: 1.0 wt% FDCA (0.202 g), Cat. Ru/support (0.1635 g), solvent=H_2_O (20 mL), *P*(H_2_)=3.1 MPa, *t*=4 h, *T*=30 °C, 50 °C, 80 °C.

**Scheme 2:**
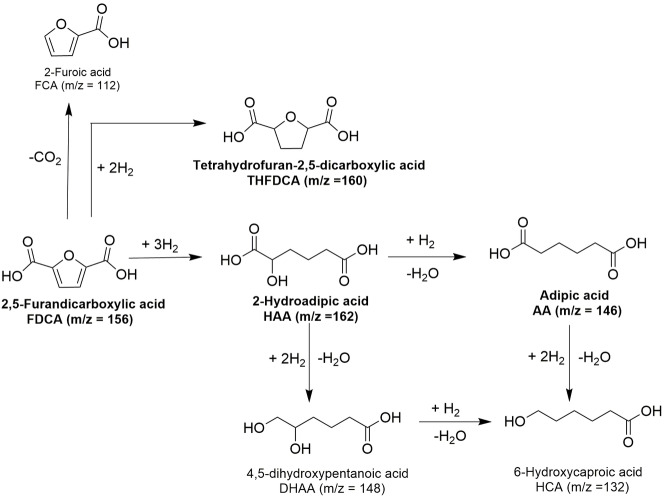
Possible products from the conversion of FDCA into AA including hydrogenation and ring‐opening processes.

The activation of FDCA by H_2_ was studied over ruthenium (Ru) supported on various oxides and carbon (C). Figure 1 exhibits the selectivity of THFDCA, HAA, and the TOF values after 4 h reaction at different temperatures (30 °C, 50 °C, and 80 °C) over Ru supported on Al_2_O_3_, ZrO_2_, TiO_2_, MnO_2_, CoO, and a commercial 5 wt% Ru/C. The Ru loading was confirmed by ICP analysis, where the Ru loading is ranged in 1.8–3.3 wt% (see the Supporting Information, Table S1). Among the tested catalysts, Ru/Al_2_O_3_ (2.0 wt% Ru) exhibited the highest activity per Ru loading at 50 °C (Figure 1c), leading to complete conversion of FDCA. Under the same reaction condition, the Ru/CoO (3.3 wt% Ru) and Ru/ZrO_2_ (2.8 wt% Ru) catalysts showed a much lower activity, and the conversion after 4 h reached only 77.1 % and 55.1 %, respectively (Figure 1c). The result implies an almost two times higher activity of the Al_2_O_3_ catalyst compared to CoO and ZrO_2_, while MnO_2_ exhibited no activity at a temperature of 50 °C. The Ru/TiO_2_ (1.8 wt% Ru) and Ru/C (5 wt% Ru) showed a high conversion of FDCA, but it exhibited lower TOF value when compared to Ru/Al_2_O_3_ (Figure 1c). Similar trends were observed at a lower temperature of 30 °C (Figure 1a) with the highest TOF value for Ru/Al_2_O_3_. As shown in Figure 1e, at 80 °C, a complete conversion of FDCA was achieved for most of the catalysts except for Ru/MnO_2_. The formation of AA and other ring‐opening by‐products [4,5‐dihydroxypentanoic acid (DHAA), 6‐hydroxycaproic acid (HCA); Scheme 2] were observed from the hydrogenation of FDCA at 80 °C, while there were no other products at lower temperature reactions (30 °C and 50 °C, Figure 1a and Figure 1c). Besides, the decrease in HAA selectivity at a lower temperature compared with the reaction at 80 °C demonstrates that a higher temperature than 80 °C strongly favors the ring‐opening of FDCA on Ru/Al_2_O_3_ and Ru/TiO_2_.

Moreover, the stability of catalysts was studied by using ICP analysis of the supernatants after the reaction. The results show that Ru leaching was negligible in most of the samples at three different temperatures (Figure 1b, d, and f). However, manganese and cobalt exhibited significant leaching after the reaction even at very low temperatures, 30 °C (Figure 1b). The concentration of manganese and cobalt in solution progressively increases upon elevated reaction temperature at 50 °C and 80 °C. This observation is in high agreement with the low Gibbs free energy change of the hydration reaction of CoO at room temperature, which the Gibbs value of −8 kJ mol^−1^ at 298 K (Eq. (1)).^[12]^ In addition, FDCA also plays as an acidic medium during the reaction, and it leads to the subsequent dissolution reaction of Co(OH)_2_ with the low negative free energy of −70.8 kJ mol^−1^,^[12]^ suggesting the high solubility of these metals under hydrothermal conditions with the presence of FDCA. In addition, by replacing the H^+^ form of the carboxylic function group of FDCA with Na^+^, we have observed that the sodium (Na^+^) form of FDCA would not induce any color change after the reaction, indicating the advantage of FDCA salt form in preventing the metal leaching during the hydrogenation reaction.

(1) $$\mathrm{CoO}+H{}_{2}O\to \mathrm{Co}\left(\mathrm{OH}\right){}_{2}\;\left(\Delta G=-8\;\mathrm{kJ}\;\mathrm{mol}{}^{-1},\;298\;K\right)$$

(2) $$\mathrm{Co}\left(\mathrm{OH}\right){}_{2}+2H{}^{+}\to \mathrm{Co}{}^{2+}+2H{}_{2}O\left(\Delta G=-70.8\;\mathrm{kJ}\;\mathrm{mol}{}^{-1},\;298\;K\right)$$

This result indicates that the low stability of MnO_2_ and CoO could lead to the poor activity of these catalysts in the hydrogenation of FDCA in water. ZrO_2_ and TiO_2_ showed high stability under hydrogenation, but they showed slightly lower activity than Al_2_O_3_, which could be caused by the very low surface area of ZrO_2_ (Table S1). Moreover, the amount of aluminum detected in the liquid phase after the reaction was negligible, indicating the high stability and the superiority of Ru/Al_2_O_3_ for the H_2_ activation of FDCA.

**Figure 2:**
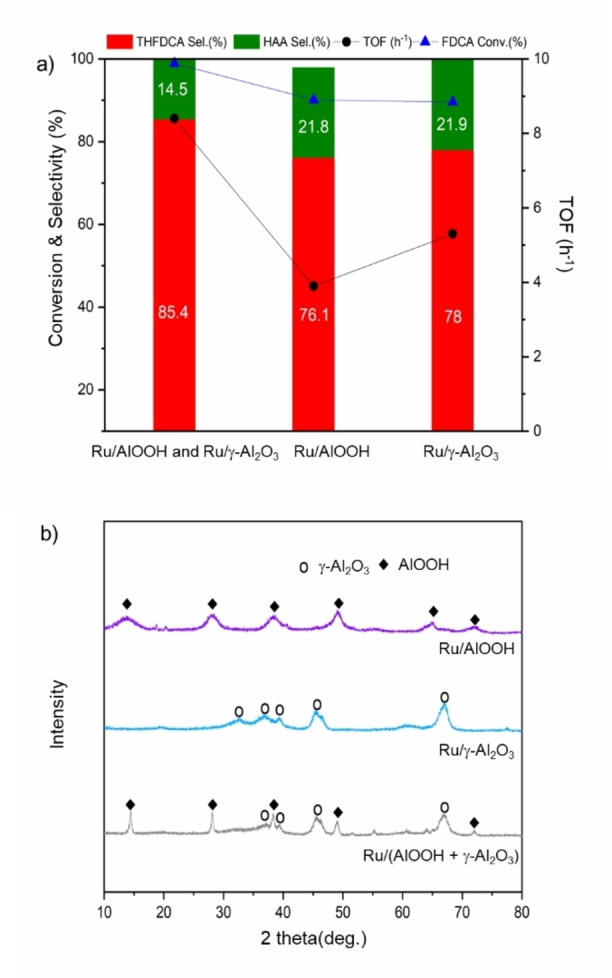
a) Effect of alumina support phase on the conversion of FDCA. Conditions: 1.0 wt% FDCA (0.202 g), Cat. Ru/alumina (0.1635 g), solvent=H_2_O (20 mL), *P*(H_2_)=3.1 MPa, *t*=4 h, *T*=50 °C. b) XRD spectra of different alumina‐supported Ru species.

Inspired by the promising results of the Ru supported Al_2_O_3_, the structural properties of Ru/Al_2_O_3_ were characterized to study the effect of its structure on the conversion of FDCA. As shown in the XRD result (Figure 2b), Al_2_O_3_ is found to consist of the mixed‐phase of AlOOH and γ‐Al_2_O_3_.

In order to see the effect of the alumina phase on the product selectivity and the dispersion of Ru, the pure AlOOH and γ‐Al_2_O_3_ were applied as support materials (Figure 2a). Among the tested catalysts, the mixed‐phase of (AlOOH+γ‐Al_2_O_3_) exhibited the complete FDCA conversion after 4 h reaction at 50 °C, with the highest TOF value of 8.4 h^−1^ (Figure 2a), while the pure AlOOH and pure γ‐Al_2_O_3_ performed lower FDCA conversion as well as much lower TOF values. Moreover, the higher THFDCA selectivity than HAA in the product distribution was observed in the case of the mixed‐phase (AlOOH+γ‐Al_2_O_3_) compared to the pure phase.

**Figure 3:**
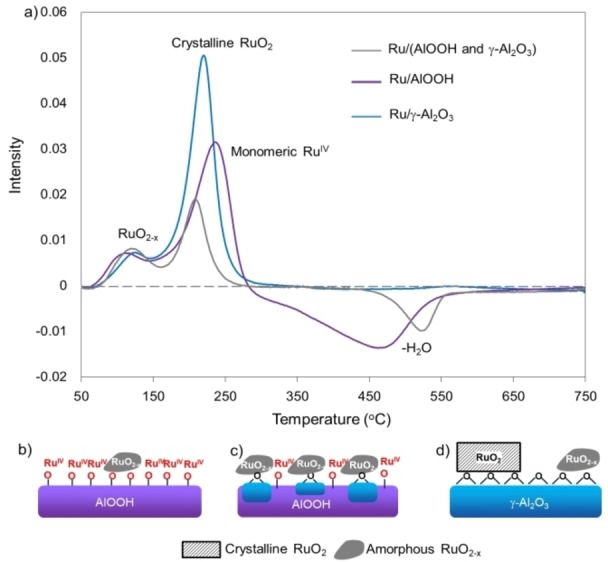
a) H_2_ temperature‐programmed reduction on different Ru/alumina catalysts. b) Strong Ru‐alumina interaction. c) Medium Ru‐alumina interaction. d) Weak Ru‐alumina interaction.

H_2_‐TPR experiments (Figure 3a) for studying the reducibility of the Ru/(AlOOH+γ‐Al_2_O_3_), Ru/AlOOH, and Ru/γ‐Al_2_O_3_ were carried out. Two reduction peaks were observed for the Ru/(AlOOH+γ‐Al_2_O_3_) at 120 °C and 200 °C, assigned to the well‐dispersed and small RuO_2‐*x*_ (dispersion: 33.5 %; Table 1, entry 1) and Ru^IV^ species, respectively.^[13]^ Ru/AlOOH also exhibited two reduction peaks, but the second one was shifted to a high temperature of 250 °C, which is attributable to the presence of monomeric surface Ru^IV^. The high surface area of Ru/AlOOH (309.52 m^2^ g^−1^) compared to Ru/γ‐Al_2_O_3_ (104.16 m^2^ g^−1^) and Ru/(AlOOH and Al_2_O_3_) (52.49 m^2^ g^−1^) would facilitate the strong interaction of Ru species with the hydroxy (OH^−^) group on the surface of AlOOH (Figure 3b). The strong interaction between the monomeric Ru^IV^ and AlOOH makes it difficult to be reduced, leading to the low dispersion of Ru NPs on the AlOOH (dispersion: 5.8 %; Table 1, entry 2). Ru/γ‐Al_2_O_3_ also showed two reduction peaks, but the second one shifted slightly to a higher temperature at 225 °C than Ru/(AlOOH+γ‐Al_2_O_3_). An increase in the integrated peak area was also observed, indicating the presence of a large portion of large size crystalline RuO_2_.^[14]^ This is likely to weaken the interaction between Ru species and γ‐Al_2_O_3_ (Figure 3d). Although the mixed‐phase of (AlOOH+γ‐Al_2_O_3_) has a much smaller surface area than the pure phases, Ru NPs are highly dispersed on the mixed phase of (AlOOH+γ‐Al_2_O_3_) (33.5 %), allowing a medium interaction between Ru species and support (Figure 3c).

**Table 1:** Physicochemical properties of Ru/alumina.

Entry	Catalyst	Alumina phase	Surface area^[a]^ [m^2^ g^−1^]	Pore volume^[b]^ [cm^3^ g^−1^]	Ru loading^[c]^ [wt %]	Ru dispersion^[d]^ [%]	Ru size^[d]^ [nm]
1	Ru/Al_2_O_3_	AlOOH and γ‐Al_2_O_3_	52.49	0.47	2.02	33.5	3.9
2	Ru/AlOOH	AlOOH	309.52	0.353	3.46	5.8	22.3^[e]^
3	Ru/γ‐Al_2_O_3_	γ‐Al_2_O_3_	104.16	0.989	2.63	20.1	6.5

[a] BET method from nitrogen adsorption isotherm. [b] BJH method from nitrogen desorption isotherm, degassing at 120 °C for 2 h before analyzing. [c] Ru loading amount was obtained by ICP analysis. [d] Ru particle size and dispersion was calculated based on H_2_ chemisorption (see the Supporting Information). [e] As the monomeric Ru^IV^ is predominant on AlOOH, the calculated diameter of Ru is an outlier.

The XPS depth profiles of Ru/AlOOH, Ru/(AlOOH+γ‐Al_2_O_3_), and Ru/γ‐Al_2_O_3_ are exhibited in Figure S2. The Al2p peak was shifted to higher binding energy in the case of Ru/AlOOH compared to Ru/(AlOOH+γ‐Al_2_O_3_) and Ru/γ‐Al_2_O_3_, which confirms of AlO−OH linkage (Figure S2b,d,f).^[15]^ Ru3p_3/2_ binding energies of around 465 eV and 463 eV were observed for Ru/AlOOH and Ru/(AlOOH+γ‐Al_2_O_3_) and maintained for different etching levels (Figure S2a,c,e), which correspond to the presence of monomeric Ru^IV^ and RuO_2‐*x*_, respectively. In the case of Ru/γ‐Al_2_O_3_, Ru3p_3/2_ peak shifted to roughly 2 eV lower at etching level=1 compared to etching level=0 while the Al2p peak shows little change. This peak shift can be attributed to the exposure of inner RuO_2‐*x*_ after etching of RuO_2_ outer surface. The lower binding energy of Ru3p_3/2_ in the Ru/(AlOOH+γ‐Al_2_O_3_) demonstrates the lower oxidation state of RuO_2‐X_, and it is in agreement with the TPR results.

From these observations, it can be concluded that during the process of Ru loading, the alumina phase has a significant effect on the generation of active Ru species for FDCA activation. The presence of hydroxy groups (OH^−^) on the surface of the mixed‐phase (AlOOH+γ‐Al_2_O_3_) limits the tendency to aggregate and thus to stabilize into small RuO_2‐*x*_ nanoparticles (NPs; 3.9 nm; Table 1, entry 1), while the OH^−^ groups rich in AlOOH phase are strengthened the interaction between Ru species and OH^−^ group on the surface of the support and are more likely to prevent the formation of Ru metal oxide, which is the active site for the reaction. On the pure γ‐Al_2_O_3_, where there is a relatively weak interaction with Ru species, it leads to the aggregation of Ru, which would induce the formation of larger crystalline RuO_2_ (6.5 nm; Table 1, entry 3).

**Figure 4:**
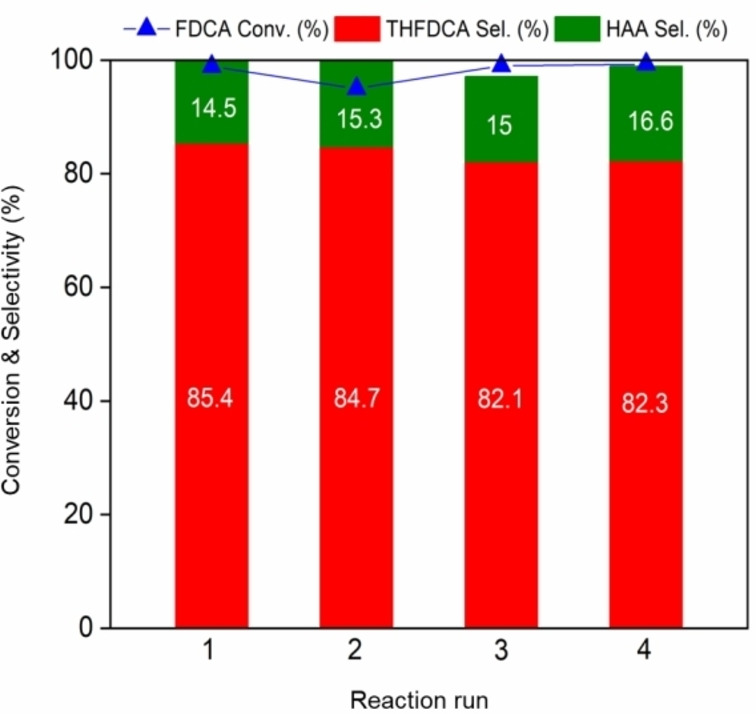
Reusability test of Ru/(AlOOH+γ‐Al_2_O_3_) on the formation of THFDCA. Conditions: 1 wt% FDCA (0.202 g), Cat. Ru/(AlOOH+γ‐Al_2_O_3_) (0.1635 g), H_2_O (20 mL), H_2_ (3.1 MPa), *T*=50 °C, 4 h.

To study the recyclability of the catalyst, Ru/(AlOOH+γ‐Al_2_O_3_) was reused in four consecutive runs under the optimal conditions (i. e., 50 °C, 4 h, 3.1 MPa of H_2_ pressure; Figures S4–S6). The catalyst was collected by centrifugation, washed with ethanol three times (100 mL), followed by drying in the oven (30 °C, 12 h) under vacuum. As shown in Figure 4, the Ru/(AlOOH+γ‐Al_2_O_3_) showed no significant loss of its activity during four cycles of catalyst re‐use. These results indicate that the recovered Ru/(AlOOH+γ‐Al_2_O_3_) retained catalytic activity after several consecutive runs. The spent catalyst maintained its original phase when comparing XRD patterns of the fresh and the used (Figure S3). Moreover, ICP analysis of the filtrate reveals that the amount of Ru and Al leached out at the end of the fourth run was only 2 % based on the initial loading, demonstrating the high stability of Ru/alumina (Table S2).

Continuous with our study, the conversion of THFDCA into AA was conducted using iodide‐containing ionic liquids (ILs) in the absence of solvents such as organic acid. The reaction time and temperature were also varied in the hydrogenolysis of THFDCA over [MIM(CH_2_)_4_SO_3_H]I, (MIM=methylimidazolium; Figures S10 and S11). From those results, a reaction temperature of 180 °C, under an H_2_ pressure of 3.4 MPa, for 2 h was considered the optimal conditions for the selective production of AA. Various ILs were tested as catalysts for the hydrogenolysis of THFDCA under optimal conditions (Table 2, entries 2–9). When typical HI was used as the catalyst, it exhibited high activity for the reaction, with 83.3 % AA yield (Table 2, entry 1), confirming the reproducibility of the previous report.^[10]^ When imidazolium iodides without −SO_3_H groups were applied as the catalysts, such as [IM]I, [MIM]I, [BIM]I, and [BMIM]I (Table 2, entries 2–5), they showed very low performance, suggesting the significant role of the −SO_3_H group as a proton source. Moreover, no improvements in the conversion were observed over the length of the alkyl chain, such as methyl ([MIM]I) and butyl group ([BIM]I) attached to the imidazolium ring (Table 2, entries 3 and 4). Besides, [MIM(CH_2_)_4_SO_3_] with −SO_3_ group but no iodide anion exhibited no activity toward AA (Table 2, entry 6), implying the critical role of iodide for furan ring‐opening.

**Table 2:** Catalyst screening for the conversion of THFDCA.^[a]^

Entry	Substrate	Catalyst	Structure	Substrate Conv. [%]	AA Yield [%]	HAA Yield [%]
1^[b]^	THFDCA	HI	HI	99.9	83.3	0
2	THFDCA	[IM]I	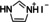	10.5	6.2	3.5
3	THFDCA	[MIM]I	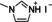	5.5	3.6	1.9
4	THFDCA	[BIM]I	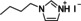	6.4	5.3	1.1
5	THFDCA	[BMIM]I	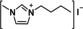	9.7	2.7	6.7
6	THFDCA	MIM(CH_2_)_4_SO_3_	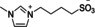	N.R	–	–
7	THFDCA	[MIM(CH_2_)_4_SO_3_H]Cl	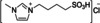	5.3	0.8	4.5
8	THFDCA	[MIM(CH_2_)_4_SO_3_H]Br	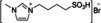	49.2	10.6	33.9
9	THFDCA	[MIM(CH_2_)_4_SO_3_H]I	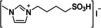	>99	98.4	0
10^[c]^	HAA	[MIM(CH_2_)_4_SO_3_H]I	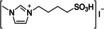	>99	99	0
11^[d]^	THFDCA + HAA	[MIM(CH_2_)_4_SO_3_H]I	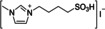	>99	>99	0

[a] THFDCA (0.165 g, 93.0 %), *n*(Sub/Cat.)=0.22, H_2_ (3.4 MPa), *T*=180 °C, *t=*2 h. [b] THFDCA (0.165 g, 93.0 %), HI 0.3 M (57 % in water, 1.0097 g), *n*(Sub/Cat.)=0.22, H_2_ (3.4 MPa), propionic acid=15 mL, *T*=180 °C, *t*=2 h. [c] HAA (0.155 g), IL (1.55 g), *n*(Sub/Cat.)=0.22, H_2_ (3.4 MPa), *T*=180 °C, *t*=2 h. [d] THFDCA 85 wt% (0.14 g), HAA 15 wt% (0.025 g), IL (1.55 g), *n*(Sub/Cat.)=0.22, H_2_ (3.4 MPa), *T*=180 °C, *t*=2 h.

On the other hand, the Hammett acidity functions (*H*_o_) of catalysts were investigated using a UV‐Vis spectrophotometer with 4‐nitroaniline as an indicator.^[16]^ The Hammett acidity functions were studied based on the difference in the absorbance before and after adding a catalyst to the 4‐nitroaniline solution. The Hammett values were calculated as Equation (3), where p*K*(In)_aq_ is the value of the indicator in water [p*K*_a_(4‐nitroaniline)≈0.99], [In] and [InH^+^] are the molar concentrations of the unprotonated and protonated form of the 4‐nitroaniline in the solution, respectively. The acid strength increases as *H*_o_ decreases.

(3) $$H{}_{o}=pK\left(In\right)aq\;-log([In]/[{InH}^{+}])$$

**Figure 5:**
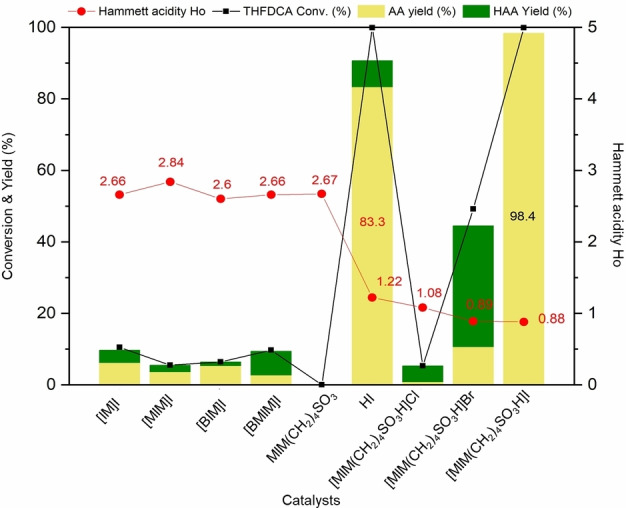
Correlation between catalytic activity and Brønsted acidity.

As shown in Figure 5, [IM]I, [MIM]I, [BIM]I, and [BMIM]I give the *H*_o_ values higher than those of other sultone‐functionalized ILs. These results are reflected in the catalytic performance, as they performed a very poor AA yields compared to other catalysts, and HAA was observed as a by‐product after the reaction (Table 2, entries 2–5). For comparison with the mineral acid catalyst, the same hydrogenolysis of THFDCA was also carried out with an equivalent concentrated HI (57 % in water) as catalyst (Table 2, entry 1).

The results demonstrate that the catalytic performance of the [MIM(CH_2_)_4_SO_3_H]I (98.4 % AA yield; Table 2, entry 9) is better than that of the concentrated HI (83.3 %, AA yield) under the same condition. In addition, mineral HI (Figure 5) shows a higher *H*_o_ than [MIM(CH_2_)_4_SO_3_H]I, indicating that the Brønsted acidity of [MIM(CH_2_)_4_SO_3_H]I is relatively stronger than that of HI. From these observations, it is obvious that Brønsted acidity strongly influences the catalytic performance during the ring‐opening, i. e., ILs having stronger acidity showed higher catalytic activities.

The halide groups′ effect was also studied by replacing the I^−^ anion in [MIM(CH_2_)_4_SO_3_H]I with Cl^−^ and Br^−^. The [MIM(CH_2_)_4_SO_3_H]Cl and [MIM(CH_2_)_4_SO_3_H]Br show almost the same H_o_ value (1.08 and 0.89) with [MIM(CH_2_)_4_SO_3_H]I (0.88; Figure 5).

**Scheme 3:**
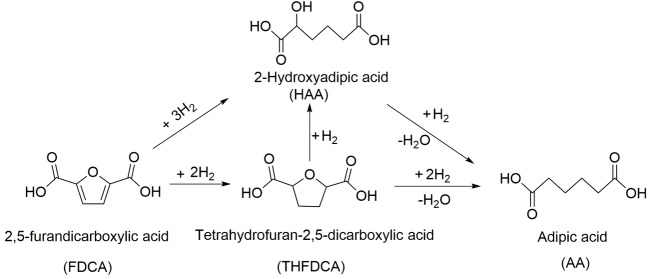
Possible pathways from FDCA into AA include hydrogenation and ring‐opening reactions with THFDCA and HAA as intermediates.

However, THFDCA conversion and HAA yield of [MIM(CH_2_)_4_SO_3_H]Cl (conv. 5.3 %, yield 0.8 %) were far lower than [MIM(CH_2_)_4_SO_3_H]Br (conv. 49.2 %, yield 10.3 %) and [MIM(CH_2_)_4_SO_3_H]I (conv. 99.9 %, yield 98.4 %). The low performance was likely due to an effect of the nucleophilicity of halides, where the nucleophilicity decreases in the sequence of I^−^>Br^−^>Cl^−^, resulting in the following activity order: [MIM(CH_2_)_4_SO_3_H]I>[MIM(CH_2_)_4_SO_3_H]Br>[MIM(CH_2_)_4_SO_3_H]Cl. On the other hand, there was no HAA was observed after the conversion of THFDCA over [MIM(CH_2_)_4_SO_3_H]I compared to other catalysts. To confirm whether HAA can be converted into AA over [MIM(CH_2_)_4_SO_3_H]I or not, reactions were performed under the same conditions with the HAA and the mixed (THFDCA and HAA) substrate (Table 2, entries 10 and 11). The results show that [MIM(CH_2_)_4_SO_3_H]I completely converted HAA into AA (Table 2, entry 10), and the same result was also observed with the mixed THFDCA and HAA as substrates. This result provides an excellent advantage in the two‐step pathway for the production of AA from FDCA, as yields of 85 % THFDCA and 15 % HAA were obtained after the first step (Figure 2a). It can be concluded that HAA is an intermediate in the hydrogenolysis of THFDCA over [MIM(CH_2_)_4_SO_3_H]I and can be converted into AA with very high selectivity due to the absence of reactive organic acid as solvent (Scheme 3), demonstrating a benefit of using [MIM(CH_2_)_4_SO_3_H]I compared to HI system.

The high activity of [MIM(CH_2_)_4_SO_3_H]I is obviously responsible for the Brønsted acidity arising from the sulfonic acid moiety. Moreover, the presence of I^−^ provided a suitable environment to act as a nucleophile for the ring‐opening step. Both are reported as crucial steps in terms of protonating and ring‐opening step in the mechanism of hydrogenolysis of THFDCA for the producing AA.^[10]^ Moreover, the AA was collected and isolated very simply after the reaction (Scheme S1), and the average isolated yield of AA was found to be 83.3 % with 98.8 % purity (Table S3).

**Figure 6:**
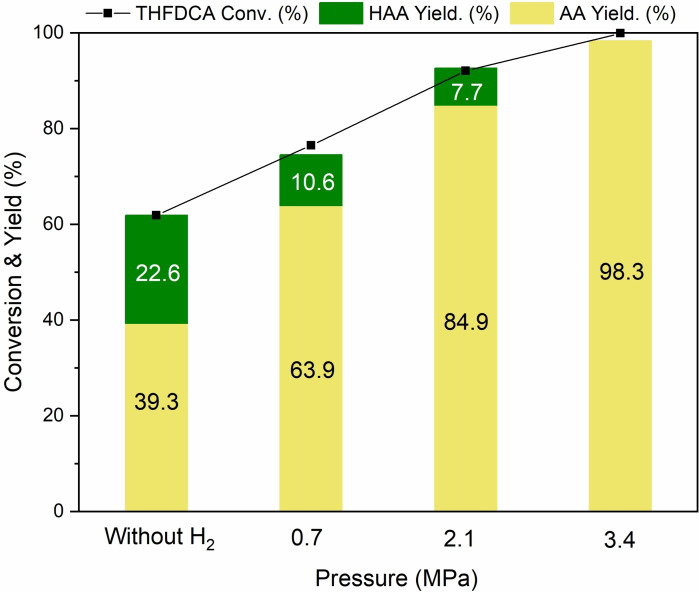
Effect of H_2_ pressure on the formation of AA over [MIM(CH_2_)_4_SO_3_H]I. Conditions: THFDCA (0.165 g, 0.958 mmol); Cat. [[MIM(CH_2_)_4_SO_3_H]I] (1.55 g), *t=*2 h, *P*=3.4 MPa, *T*=180 °C.

The influence of H_2_ pressure on the activity of [MIM(CH_2_)_4_SO_3_H]I is illustrated in Figure 6. Elevating H_2_ pressure from 0.7 to 2.1 MPa led to a gradual increase in THFDCA conversion (76.5 % to 92.1 %) and AA yield (63.9 % to 84.9 %) with a small amount of HAA as an intermediate. A further increase in H_2_ pressure to 3.4 MPa resulted in the complete conversion of THFDCA along with 98.3 % of AA yield. Interestingly, when the reaction was performed in the absence of an H_2_, [MIM(CH_2_)_4_SO_3_H]I performed a 39.3 % yield of AA. This result suggests that a dissociation of [MIM(CH_2_)_4_SO_3_H]I would occurr, leading to zwitterionic IL, (MIM(CH_2_)_4_SO_3_) and an HI (Eq. (4)), and the HI would subsequently decompose to act as an internal hydrogen source for producing HAA (Eq. (5)) and iodine molecule.

(4) $$\left[\mathrm{MIM}\left(\mathrm{CH}{}_{2}\right){}_{4}\mathrm{SO}{}_{3}H\right]I\;\overset{\leftarrow }{\to }\;\mathrm{MIM}\left(\mathrm{CH}{}_{2}\right){}_{4}\mathrm{SO}{}_{3}+\mathrm{HI}$$

(5) $$2\mathrm{HI}\overset{\leftarrow }{\to }H{}_{2}+I{}_{2}$$

(6) $$2\mathrm{MIM}\left(\mathrm{CH}{}_{2}\right){}_{4}\mathrm{SO}{}_{3}+H{}_{2}+I{}_{2}\overset{\leftarrow }{\to }2\left[\mathrm{MIM}\left(\mathrm{CH}{}_{2}\right){}_{4}\mathrm{SO}{}_{3}H\right]I$$

**Scheme 4:**
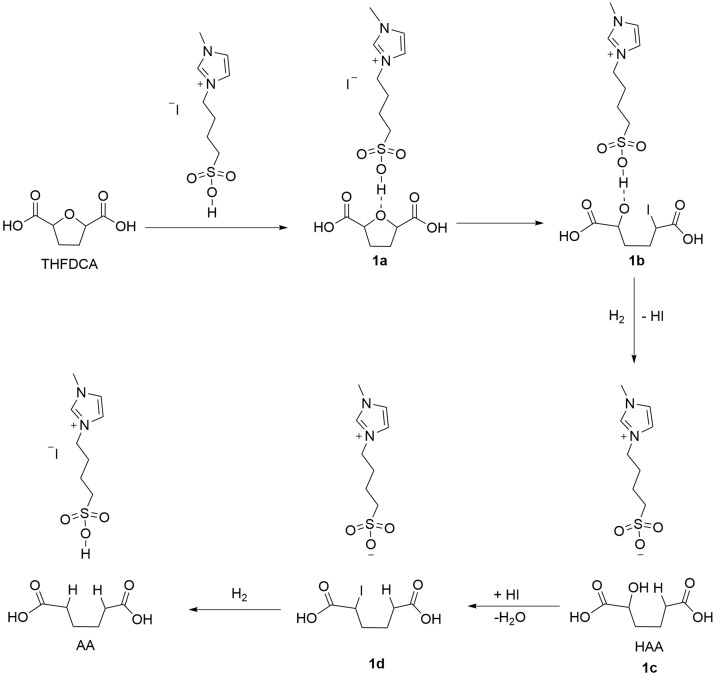
Proposed mechanism of the production of THFDCA over [MIM(CH_2_)_4_SO_3_H]I.

Such a mechanistic investigation is well described in the literature.[^10^, ^17^] The first step would be a protonation with a proton from [MIM(CH_2_)_4_SO_3_H]I to form an oxonium intermediate **1 a** (Scheme 4), and subsequent C−O bond cleavage by a nucleophile (I^−^) to generate **1 b**. In the presence of a hydrogen source, which can come from the internal source [Eq. (4)] or the external source, **1 c** (HAA) is generated through hydrogenolysis of the C−I bond. Accordingly, a fast iodide substitution leads to **1 d** [2‐iodohexanedioic acid (IAA)]. Finally, the subsequent hydrogenolysis of **1 d** produces the desired AA.

Under H_2_ pressure (external hydrogen source), then [MIM(CH_2_)_4_SO_3_H]I could be regenerated driven by (Eq. (6)). Therefore, the high catalytic performance of [MIM(CH_2_)_4_SO_3_H]I can be ascribed to an efficient IL system that conveys hydrogen from external to internal through a series of stepwise reactions as described in Equation (4)–(6). As a result, [MIM(CH_2_)_4_SO_3_H]I provides a Brønsted acidic proton from −SO_3_H, the high nucleophilicity of I^−^, and an ability to generate internal H_2_, and thus this sequence proceeds well for the reaction. Considering that excess [MIM(CH_2_)_4_SO_3_H]I was applied for this reaction, this IL system can be described as good reaction medium as well as an efficient catalyst.

**Figure 7:**
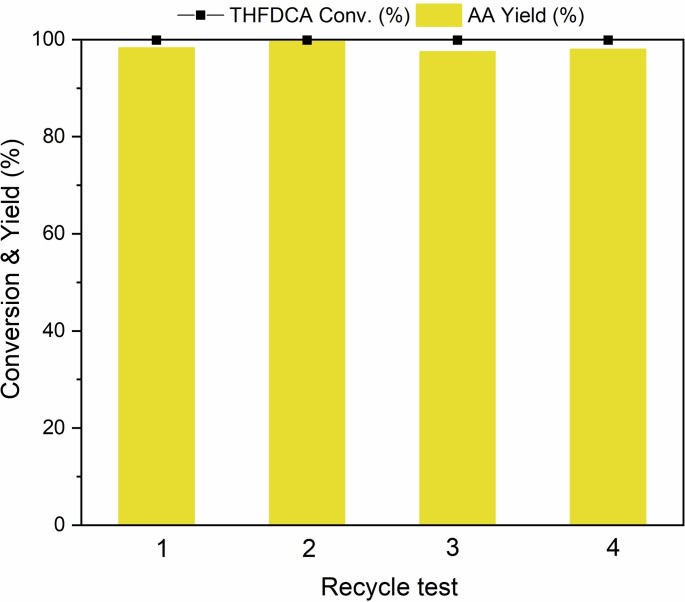
Reusability test of [MIM(CH_2_)_4_SO_3_H]I. Conditions: THFDCA (0.165 g, 93 %), *n*(Sub/Cat.)=0.22, H_2_=3.4 MPa, *T*=180 °C, *t*=2 h.

To study the recyclability of [MIM(CH_2_)_4_SO_3_H]I, the used catalyst was collected after the first run and was regenerated by treating with HI solution before applying to the next cycle (Eq. (6) and Scheme S1). The regenerated catalyst was reused for the successive reactions with a fresh charge of THFDCA under the same experimental conditions. As shown in Figure 7, [MIM(CH_2_)_4_SO_3_H]I retains most of its original activity even after four consecutive runs, indicating that [MIM(CH_2_)_4_SO_3_H]I is highly recyclable. The ^1^H NMR spectrum of the spent IL (Figure S18) showed that the regenerated IL maintained its original structure after the fourth run.

## Conclusions

In summary, we have shown that FDCA can be converted into AA with a 99 % yield by a two‐step process. First, the hydrogenation and hydrogenolysis of FDCA to THFDCA and HAA was conducted by using various oxide‐supported Ru catalysts. Among them, Al_2_O_3_, which consists of AlOOH and γ‐Al_2_O_3_ phases, was found to be an efficient support for Ru in this reaction. H_2_ chemisorption and H_2_‐TPR analysis on Ru/Al_2_O_3_ demonstrated the significant effect of the mixed‐phase (AlOOH+γ‐Al_2_O_3_) on improving the Ru distribution and its nanoparticle size, which leads to an enhancement in the conversion of FDCA. The Ru/Al_2_O_3_ gave a complete conversion of FDCA into THFDCA and HAA under 3.1 MPa H_2_ at 50 °C for 4 h. ICP analysis of the filtrate after the reaction showed a negligible amount of Ru and Al, indicating the high stability of Ru/Al_2_O_3_. Furthermore, the Ru/Al_2_O_3_ maintained high activity toward THFDCA for four consecutive runs, retaining its structure after the reaction. A Brønsted acidic ionic liquid system, [MIM(CH_2_)_4_SO_3_H]I, was developed for the selective production of AA from THFDCA and HAA in the absence of any organic acid solvents. [MIM(CH_2_)_4_SO_3_H]I showed higher activity than the conventional HI system, delivering 99 % yield of AA at complete conversion of THFDCA and HAA. Hammett's acidity measurement exhibited the strong Brønsted acidity of [MIM(CH_2_)_4_SO_3_H]I, which contributes to the excellent performance in the ring‐opening of THFDCA. [MIM(CH_2_)_4_SO_3_H]I exhibited fairly good activity, even in the absence of hydrogen pressure, suggesting dissociation of [MIM(CH_2_)_4_SO_3_H]I, formation of HI, and subsequent decomposition of HI into H_2_ and I_2_. The high catalytic activity of [MIM(CH_2_)_4_SO_3_H]I can be ascribed to an efficient IL system that conveys hydrogen from external to internal through a series of reactions such as aforementioned dissociation and decomposition and a role of I^−^ as a nucleophile. Furthermore, this reaction system of [MIM(CH_2_)_4_SO_3_H]I provides a simple product separation process as well as an IL‐reuse strategy.

Overall, we developed a recyclable reaction medium and a multicatalytic system for the production of AA from FDCA that avoids the use of noble metals and corrosive inorganic and organic acids. Therefore, this strategy can be evaluated as economically and environmentally viable for the mass production of AA from biomass‐derived chemicals.

## Experimental Section

### Materials

Al_2_O_3_ (powder, 99.9 %), 4‐butane sultone (liquid, 99 %), 1‐methylimidazole (liquid, 99 %), and HI (57 wt% aqueous solutions) were purchased from Alfa Aesar. RuCl_3_ ⋅ *x*H_2_O (powder, 99.8 %), and NaBH_4_ (powder, 98 %) were obtained from DeaJung Co., Ltd. ZrO_2_ (powder, 99 %), TiO_2_ (powder, 99.7 %), MnO_2_ (powder, 99 %), CoO (powder, 99.9 %), and Ru/C (powder, 5 wt% Ru) were purchased from Sigma‐Aldrich. 2,5‐furan dicarboxylic acid (FDCA, 99 %) and tetrahydrofuran‐2,5‐dicarboxylic acid (THFDCA, 93 %) were purchased from Angene International Co., Ltd. Adipic acid (AA, 99 %) was obtained from Sigma. Hydroxyadipic acid (HAA) was purchased from Habo Hong Kong Co., Ltd. The above‐authenticated samples were used as received.

### Catalyst preparation

#### Ruthenium loading onto support materials

Typically, support (1.0 g) and RuCl_3_ ⋅ *x*H_2_O (0.085 g; M.W. 207.43) were placed together in a 100 mL round bottom flask containing DI water (20 mL). The resulting mixture was stirred for 12 h. Then, an aqueous solution of NaBH_4_ (10 times higher than RuCl_3_ ⋅ *x*H_2_O) was added dropwise to the reaction mixture with constant stirring for 24 h at room temperature. Finally, the obtained catalyst was separated by filtration, washed with ethanol five times (100 mL), and dried under vacuum for 12 h.

#### Iodide‐based IL preparation

For the preparation of [MIM‐(CH_2_)_4_HSO_3_]I, 1,4‐butane sultone (12.0 g) was dissolved in ethyl acetate (EA; 50 mL), then 1‐methylimidazole (8.2 g) was added to the solution at 50 °C. The mixture was stirred for 12 h, and the resultant mixture was filtered to get a white precipitate [MIM‐BS]. The precipitate was washed with EA three times and dried at 100 °C for 2 h to get [MIM‐BS] as a white solid. After that, of [MIM‐BS] (4.0 mmol) was dissolved in water (5 mL), and hydroiodic acid (HI; 4.0 mmol) was added slowly at room temperature. The mixture was stirred at 90 °C for 4 h. Then, the solvent was removed under vacuum at 90 °C to get [MIM‐(CH_2_)_4_HSO_3_]I as a yellow viscous liquid. The preparation process for other ILs is described in the Supporting Information.

### Catalyst characterization

The powder X‐ray diffraction (XRD) measurements were carried out at room temperature on a range from 10° to 80° on Bruker D505 powder diffractometer using a Cu anode as the X‐ray source to determine the phase of alumina in the Ru/alumina. The nitrogen adsorption/desorption analysis was operated on a Belsorp‐II (Japan). Prior for analyzing, each sample was degassed at 200 °C for 30 min under vacuum. The specific surface area was calculated according to the Brunauer‐Emmett‐Teller (BET) method. The ruthenium contents of the Ru/metal oxides samples were determined by using Inductively coupled plasma optical emission spectroscopy (ICP‐OES) analysis on an ICP‐ Agilent 7900 after each sample was entirely dissolved in the mixture of HNO_3_/HCl solution. Field‐emission scanning electron microscope (FE‐SEM) images of Ru/metal oxides were taken using a JEOL JSM‐6701F/INCA system at 15kv with platinum coating on samples. The X‐ray photoelectron spectroscopy (XPS) was measured on a K‐alpha Thermo Fisher spectrometer with 225 W of AlK_α_ radiation to determine the chemical states of Ru/metal oxide samples. The pristine XPS spectra of Ru3p and Al2p were present without modification as the C1s peak overlaps with the Ru3d peak. The XPS depth profile analysis was conducted after 120 s etching with Ar^+^ ion accelerated at 3 keV. Temperature programmed reduction by hydrogen (H_2_‐TPR) was conducted on a Micromeritics Autochem II chemisorption analyzer with a thermal conductivity detector (TCD). Before the test, the sample was pretreated in Argon (50 ml/min) at 200 °C. Then the catalyst was treated in a 10 % H_2_/Ar (50 ml min^−1^) gas mixture at 200 °C. The hydrogen consumption was estimated by TCD. ^1^H and ^13^C nuclear magnetic resonance (NMR) spectra of the ionic liquids were collected by a nuclear magnetic resonance (Bruker Avance III 300 MHz) spectrometer. Hammett acidity functions (*H*_o_) of catalysts were investigated using a UV‐Vis spectrophotometer with 4‐nitroaniline as an indicator.

### Hydrogenation of FDCA

The hydrogenation of FDCA was conducted in a 50 mL stainless‐steel high‐pressure reactor equipped with a stirrer, a thermocouple, and a pressure controller. For each experiment, FDCA (0.202 g), catalyst (0.1635 g), and water (20 mL) were introduced into the reactor. The system was purged with H_2_ three times to remove air at room temperature, then further charged with 3.1 MPa of H_2_ at 50 °C and maintained during the reaction. After the reaction, the reactor was cooled to room temperature, followed by depressurization to unseal.

Reusability experiments of the solid catalyst were performed in the same process but after removing the liquid solution by centrifugation. The solid catalyst was washed with ethanol three times (30 mL), dried in a vacuum oven, then introduced into the reactor with a fresh amount of FDCA for a subsequent catalytic cycle.

After filtering the catalyst, the liquid mixture was analyzed by HPLC (Agilent 1260 Infinity II) equipped with a Biorad HPX‐87X column (4.6×210 mm) and detected with the UV wavelength of 210 nm at 60 °C. The mobile phase consisted of 2.7 mM H_2_SO_4_ at a flow rate of 0.6 mL min^−1^, and the injection volume was 10 μL. The product yield and reactant conversion were calculated based on the calibration curves of the FDCA, THFDCA, AA, and HAA standards with the adding of DMF as an external standard [Eqs. (7)–10]:

(7) $$FDCA\;conversion\;\left(\%\right)=\;\frac{FDCA\;consumed\;\left(mmol\right)}{FDCA\;substrate\;charged\;\left(mmol\right)}\times 100$$

(8) $$THFDCA\;selectivity\;\left(\%\right)=\;\frac{THFDCA\;produced\;\left(mmol\right)}{FDCA\;substrate\;consumed\;\left(mmol\right)}\times 100$$

(9) $$HAA\;selectivity\;\left(\%\right)=\;\frac{HAA\;produced\;\left(mmol\right)}{FDCA\;substrate\;consumed\;\left(mmol\right)}\times 100$$

(10) $$AA\;selectivity\;\left(\%\right)=\;\frac{AA\;produced\;\left(mmol\right)}{FDCA\;substrate\;consumed\;\left(mmol\right)}\times 100$$

TOF values were calculated based on Equation 11:

(11) $$TOF\;\left(h^{-1}\right)=\frac{\left(mmol\;of\;produced\;THFDCA\right)}{\left(mmol\;of\;Ru\;loading\;\times h\right)}$$

### Ring‐opening of THFDCA to AA

Hydrodeoxygenation reaction of tetrahydrofuran‐2,5‐dicarboxylic acid was conducted in a 50 mL stainless‐steel Hastelloy high‐pressure reactor equipped with a stirrer, a thermocouple, and a pressure gauge. In a typical experiment, 0.165 g of THFDCA, 1.55 g of catalyst, and 15 mL of solvent were introduced into the reactor. The reactor was heated to the desired temperature, where it was maintained till the completion of the reaction. The system was then charged with the desired pressure of 3.4 MPa H_2_ and stirred at a speed of 450 rpm. After completion, the system was immediately cooled down in the cooling bath and depressurized. The reaction solution was analyzed by HPLC (Agilent 1260 series) equipped with an HPX‐87X column (4.6×250 mm) and detected with the UV wavelength of 210 nm at 35 °C. The mobile phase consisted of 0.1 wt% HCOOH at a flow rate of 0.6 mL min^−1^, and the injection volume was 10 uL. The product yield and reactant conversion were calculated based on the calibration curves of the THFDCA, AA, and HAA standards [Eqs. (12)–14]:

(12) $$THFDCA\;conversion\;\left(\%\right)=\;\frac{THFDCA\;consumed\;\left(mmol\right)}{THFDCA\;substrate\;charged\;\left(mmol\right)}\times 100$$

(13) $$AA\;yield\;\left(\%\right)=\frac{AA\;produced\;\left(mmol\right)}{THFDCA\;substrate\;charged\;\left(mmol\right)}\times 100$$

(14) $$HAA\;yield\;\left(\%\right)=\;\frac{HAA\;produced\;\left(mmol\right)}{THFDCA\;substrate\;charged\;\left(mmol\right)}\times 100$$

## Conflict of interest

The authors declare no conflict of interest.

1

## Supporting information

As a service to our authors and readers, this journal provides supporting information supplied by the authors. Such materials are peer reviewed and may be re‐organized for online delivery, but are not copy‐edited or typeset. Technical support issues arising from supporting information (other than missing files) should be addressed to the authors.

Supporting Information

## Data Availability

The data that support the findings of this study are available in the supplementary material of this article.
