# Supplementary material for: Efficient Production of Adipic Acid by a Two‐Step Catalytic Reaction of Biomass‐Derived 2,5‐Furandicarboxylic Acid
Source: ChemSusChem. 2022 Apr 1;15(10):e202200375. doi: 10.1002/cssc.202200375 (PMC9323459; doi:10.1002/cssc.202200375)
Supplement: Supplementary file 1 — Supporting Information [file CSSC-15-0-s001.pdf]

ChemSusChem

Supporting Information

Efficient Production of Adipic Acid by a Two-Step Catalytic Reaction of Biomass-Derived 2,5-Furandicarboxylic Acid

Anh Vy Tran+, Seok-Kyu Park+, Hye Jin Lee, Tae Yong Kim, Younhwa Kim, Young-Woong Suh, Kwan-Young Lee, Yong Jin Kim,* and Jayeon Baek*© 2022 The Authors. ChemSusChem published by Wiley-VCH GmbH. This is an open access article under the terms of the Creative Commons Attribution License, which permits use, distribution and reproduction in any medium, provided the original work is properly cited.

Contents:

Section 1: Synthesis of catalysts 2

Preparation of Iodide-based ionic liquids 2

Section 2: Conversion of FDCA to THFDCA and HAA 3

Catalyst characterization 3

ICP analysis of different Ru loadings onto metal oxides 3

FE-SEM analysis of Ru/alumina with different phase of alumina 5

XPS analysis of of Ru/alumina with Ru 3p and Al 2p spectra at etching level = 0, 1, 5. 6

XRD spectra of fresh and spent Ru/(AlOOH and γ-Al_2O_3) catalyst 7

Effect of reaction parameters on the formation of THFDCA 8

Effect of FDCA concentration on the formation of THFDCA over Ru/Al_2O_3 11

Effect of calcination temperature on AlOOH support and its effect on the conversio n of FDCA 13

Section 3: Conversion of THFDCA to AA 14

Effect of reaction parameters on the formation of AA 14

AA isolation process and regeneration of spent catalyst (IL) after the reaction 16

Section 4: Two-step pathway for producing AA from a high concentration of FDCA. 19

Section 5: ^{1}H-NMR and ^{13}C-NMR spectra 20

Section 6: 30

References 30

Section 1: Synthesis of catalysts

Preparation of Iodide-based ionic liquids

For the preparation of MIM-BS: 12 g of 1,4-butane sultone was dissolved in 50 mL Ethyl acetate (EA), then 8.2 g 1-methylimidazole was added to the solution at 50 °C. The mixture was stirred for 12 h, and the resultant mixture was filtered to get a white precipitate [MIMBS]. The precipitate was washed with EA three times and dried at 100 °C for 2 h to get [MIM-BS] as a white solid.

[MIM-$(CH_2)_4HSO_3$]Br: 5 g [MIM-BS] was dissolved in 5 mL water, and 3.86 g hydrobromic acid (48% in water, MW 80.9) was added slowly at room temperature. The mixture was stirred at 90 °C for 4 h. After that, the water was removed under vacuum at 90 °C to get [MIM-$(CH_2)_4HSO_3$]Br as a yellow viscous liquid.

[MIM-$(CH_2)_4HSO_3$]Cl: 5 g [MIM-BS] was dissolved in 5 mL water, and 2.32 g hydrochloric acid (36 % aqueous, MW 36.5) was added slowly at room temperature. The mixture was stirred at 90 °C for 4 h. After that, the water of the mixture was removed under vacuum at 90 °C to get [MIM-$(CH_2)_4HSO_3$]Cl as a yellow viscous liquid.

[IM]I: 20 mmol of imidazole and acetonitrile were charged into a 25 mL round bottom flask. After that, it was kept in an ice bath to maintain 0 °C. An equimolar amount of HI (20 mmol) was added dropwise into the flask under vigorous stirring. The mixture was stirred for another 1 h at 0 °C followed by 2 h stirring at room temperature. The product was then washed with diethyl ether three times. The final product was dried in a rotary evaporator for 2 h under reduced pressure.

[MIM]I: The procedure was followed [IM]I preparation except for 20 mmol of methyl imidazole and acetonitrile were charged into 25 mL round bottom flask.

[BIM]I: The procedure was followed [IM]I preparation except for 20 mmol of butyl imidazole and acetonitrile were charged into 25 mL round bottom flask.

Section 2: Conversion of FDCA to THFDCA and HAA

Catalyst characterization

ICP analysis of different Ru loadings onto metal oxides

Table S1. ICP analysis of different Ru loading onto metal oxides.

No.	Samples	BET surface area ($m^2.g^{-1}$)	Ru loading wt.% (ICP analysis)
1	Ru/ZrO_2	3.73	2.8
2	Ru/Al_2O_3	52.49	2.0
3	Ru/TiO_2	54.93	1.8
4	Ru/MnO_2	74.06	2.9
5	Ru/CoO	58.59	3.3

Table S2. ICP analysis of the supernatant solution after 4th hydrogenation reaction of FDCA over Ru/alumina.

Element	After 4th recycle test (ppm)	% metal loss
Ru	4.1	2%
Al	93.1	2%

H_2 chemisorption analysis

Ru NPs size and its dispersion were calculated as the below equations[1]:

$$D(\%) = \frac{\eta \times S \times M}{w} \times 10^2$$

$$d(nm) = \frac{f \times w}{\rho \times \eta \times N(A) \times S \times A} \times 10^5$$

Where:

D : Dispersion (%).

η : Desorbed H_2 (mol/g).

S : Stoichiometric factor for H_2 chemisorption (metal mole/gas mole =2).

M : Atomic mass of Ru (101.07).

w : Weight percentage of metal (%).

d(nm) : Particle size.

f : Geometric shape factor (6 for spherical particles).

ρ : Density of Ru.

N(A) : Avogadro number (6.023×10^{23}).

A: Area occupied by the Ru surface atom.

FE-SEM analysis of Ru/alumina with different phase of alumina

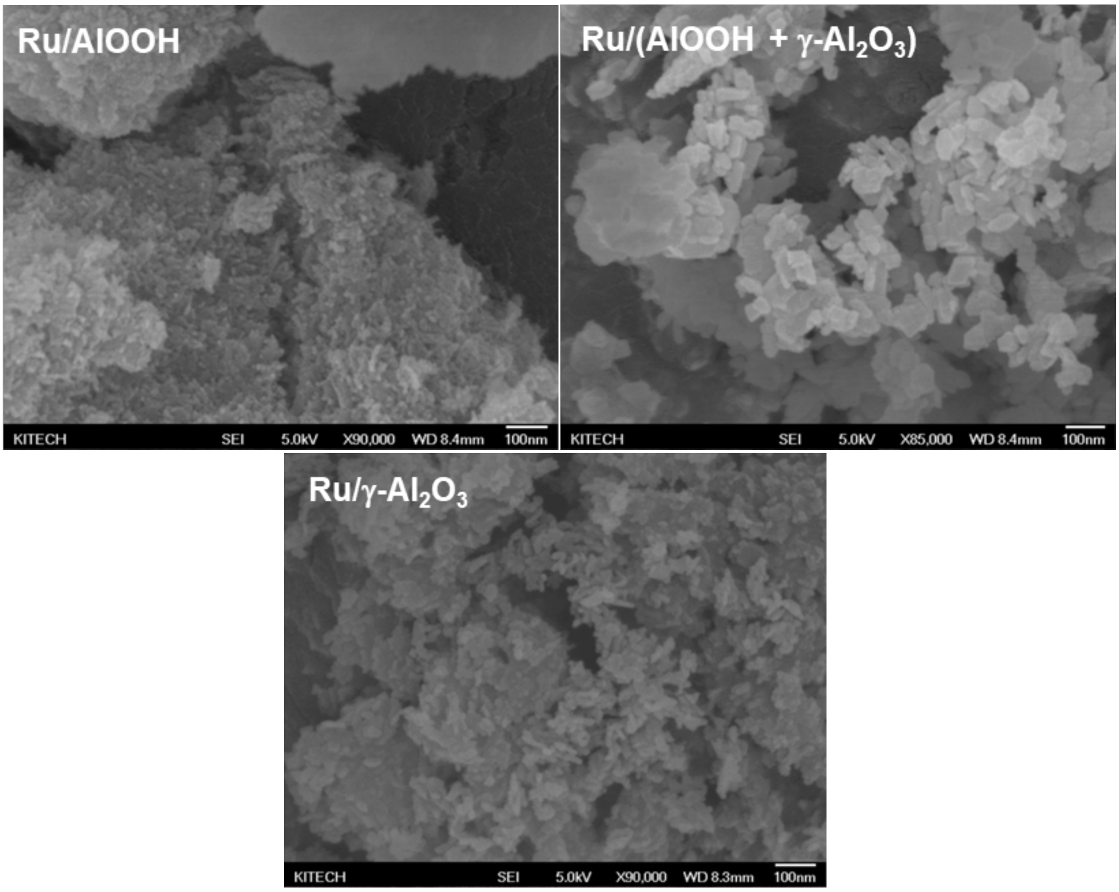

Figure S1. FE-SEM images of Ru/alumina with different phases of alumina.

XPS analysis of of Ru/alumina with Ru 3p and Al 2p spectra at etching level = 0, 1, 5.

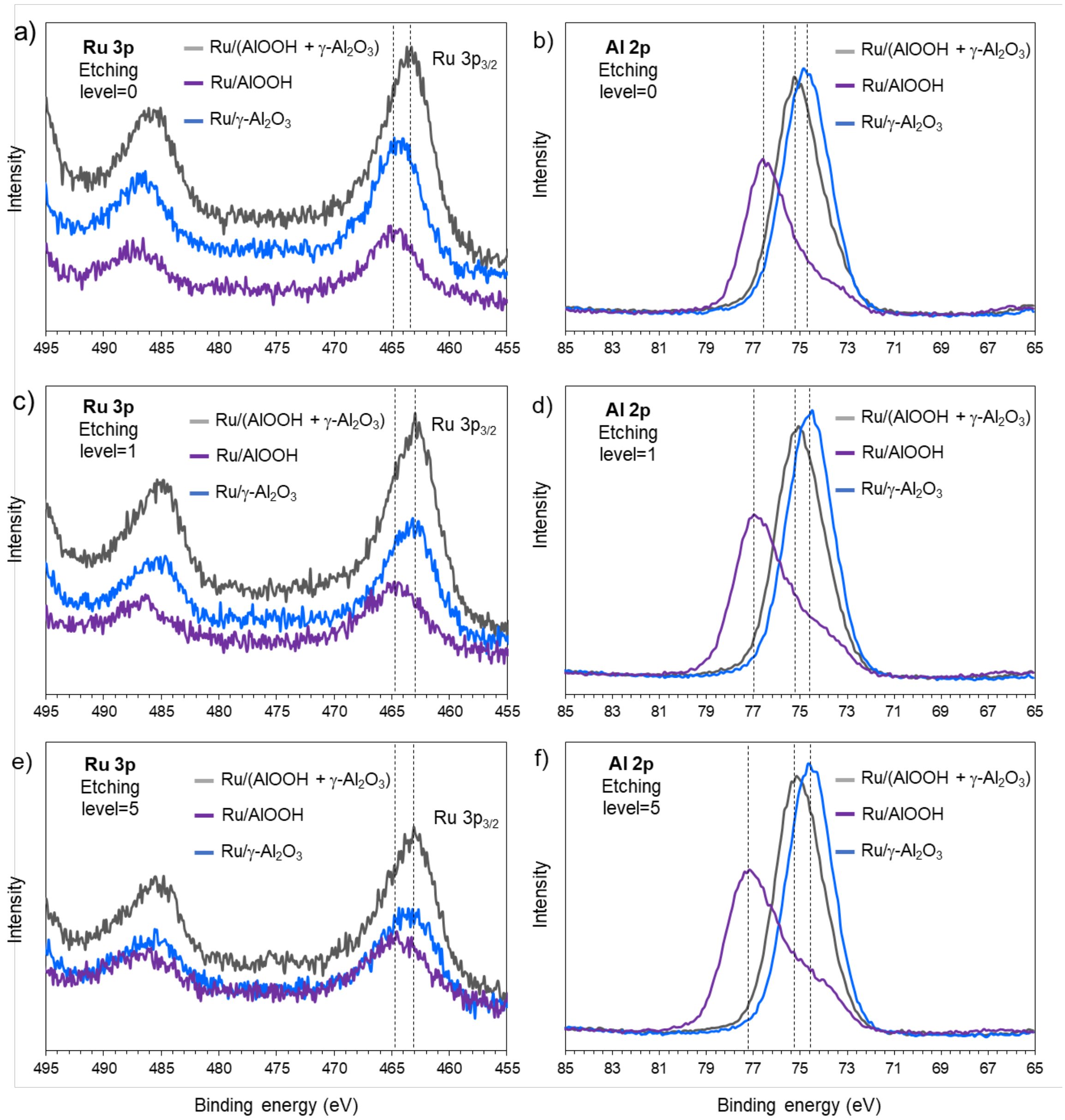

Figure S2. Depth-profiles of XPS analysis of Ru/alumina with different phases of alumina: a) Ru 3p at etching level = 0, b) Al 2p at etching level = 0, b) Ru 3p at etching level = 1, d) Al 2p at etching level = 1, e) Ru 3p at etching level = 5, Al 2p at etching level = 5.

XRD spectra of fresh and spent Ru/(AlOOH and γ-Al_2O_3) catalyst

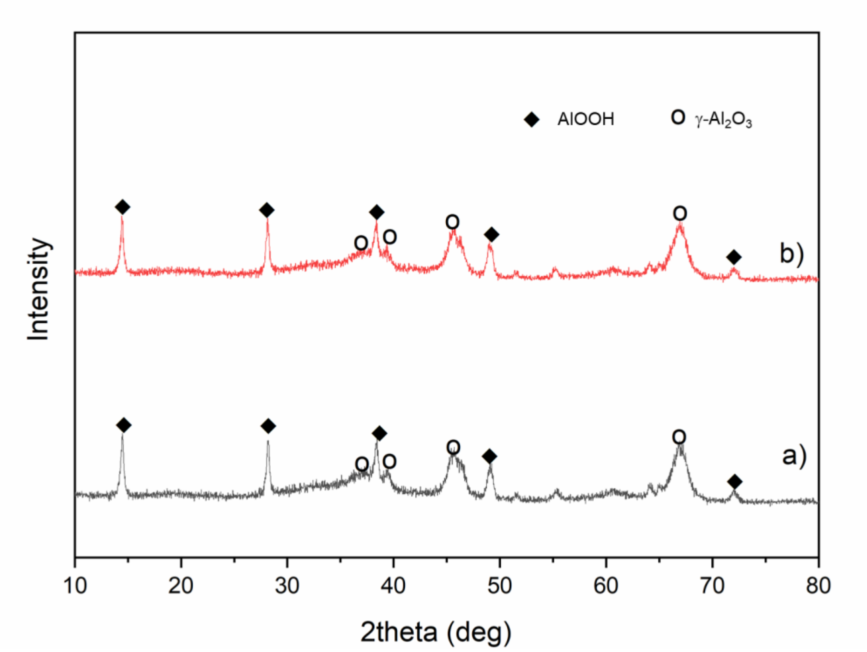

Figure S3. XRD spectra of Ru/(AlOOH and γ-Al_2O_3) (a) fresh and (b) after 4th recycle test.

Effect of reaction parameters on the formation of THFDCA

The reaction parameters were also varied in the hydrogenation of FDCA over Ru/Al_2O_3 in order to be optimized. As depicted in Figure S4, by increasing temperature from 30 °C to 50 °C, a significant increase in FDCA conversion was observed (38.1% to 99.9%) but almost no change in the THFDCA selectivity (83.5% to 85.4%). The conversion of FDCA remained constant when elevating the temperature to 80 °C. However, further increasing the reaction temperature (50 °C to 80 °C) led to a decrease in the THFDCA selectivity and an increase in the HAA selectivity. In addition, 3% of AA selectivity was obtained at a temperature of 80 °C. It indicates that more THFDCA was converted into HAA and AA, ring-opening products of the FDCA hydrogenation reaction.

This result revealed that the ring-opening of FDCA may not happen below 80 °C, but it was accelerated by increasing the reaction temperature. From these results, 50 °C was the optimal temperature for the hydrogenation of FDCA in this reaction system.

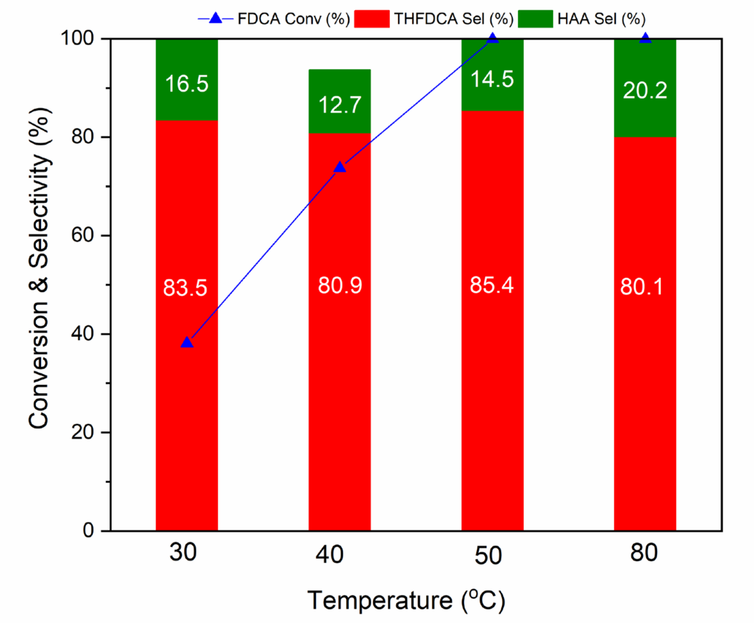

Figure S4. Effect of reaction temperature on the formation of THFDCA over Ru/Al_2O_3.

Conditions: 1 wt.% FDCA (0.202 g), Cat. Ru/Al_2O_3 (0.1635 g), H_2O 20 mL, H_2 3.1 MPa, 4 h.

As shown in Figure S5, at the initial stage of the hydrogenation (1 h), FDCA was quickly converted to THFDCA with a selectivity of 78%. The conversion of FDCA was increased gradually and was fully converted into THFDCA with a selectivity of 85.4% at 4 h. After complete hydrogenation, a longer reaction time (6 h) strongly changed the product distribution; the THFDCA selectivity decreased to 75.5%, whereas the HAA selectivity increased to 22.5%. This phenomenon suggested that THFDCA could be converted into HAA through ring-opening in our reaction system.

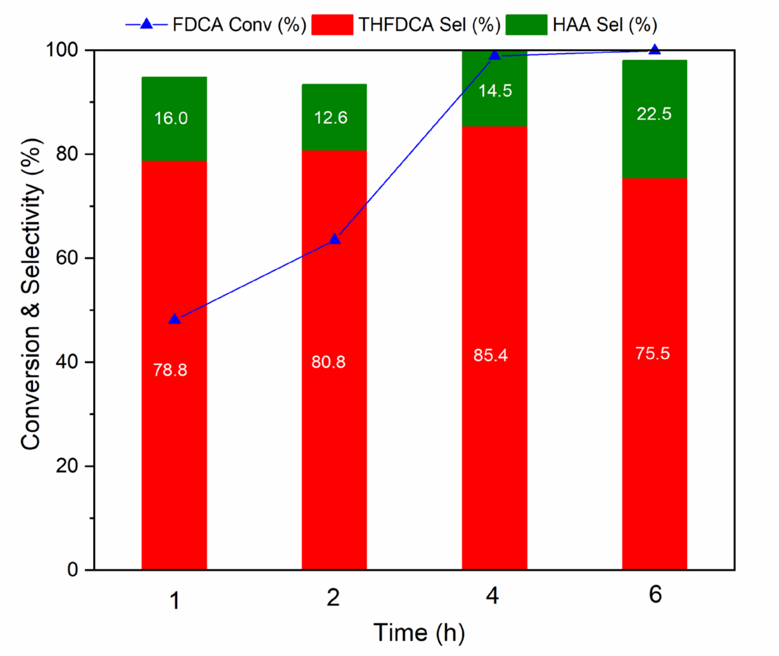

Figure S5. Effect of reaction time on the formation of THFDCA over Ru/Al_2O_3

Conditions: 1 wt.% FDCA (0.202 g), Cat. Ru/Al_2O_3 (0.1635 g), H_2O 20 mL, H_2 3.1 MPa, T = 50 °C.

Figure S6 shows the influence of the H_2 pressure on the selectivity of the products. An increase in the THFDCA selectivity and conversion of FDCA occurred when the H_2 pressure was elevated from 1.7 to 3.8 MPa, indicating that FDCA was converted mainly to THFDCA at relatively moderate pressure (3.1 MPa). When comparing the reactions carried out at 3.1 and 3.8 MPa, the conversion of FDCA kept constant, and the THFDCA selectivity did not change notably. Therefore, a moderate pressure of 3.1 MPa was preferred to maintain the reaction activity and inhibit the complete hydrogenation of the furan ring.

On the basis of the influence of the reaction conditions on the selectivity of THFDCA, we chose a reaction temperature of 50 °C, an H_2 pressure of 3.1 MPa, and a reaction time of 4 h as the optimal conditions for the selective production of THFDCA from FDCA.

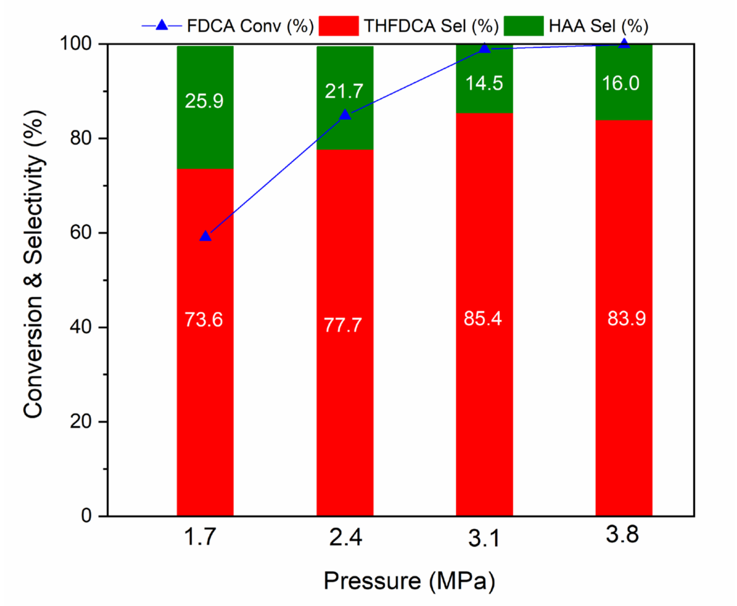

Figure S6. Effect of H_2 pressure on the formation of THFDCA over Ru/Al_2O_3.

Conditions: 1 wt.% FDCA (0.202 g), Cat. Ru/Al_2O_3 (0.1635 g), H_2O 20 mL, T = 50 °C, t = 4 h.

Effect of FDCA concentration on the formation of THFDCA over Ru/Al_2O_3

The impact of FDCA concentration on the formation of THFDCA was studied by using Ru/Al_2O_3 as the main catalyst. A longer reaction time (8 h, 20 h) was applied in the case of 5 wt.%, and 10 wt.% FDCA, respectively. As expected, prolonging reaction time leads to complete conversion of FDCA at a higher concentration of FDCA (Figure S7).

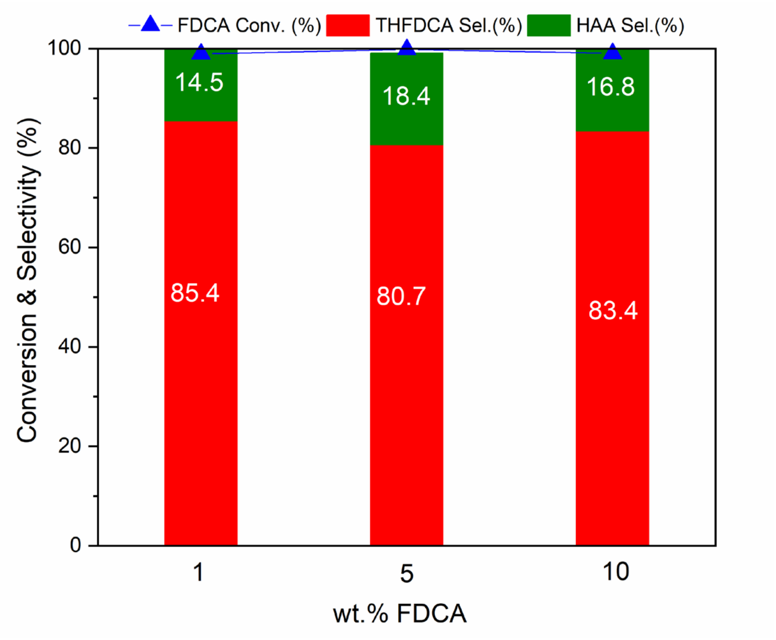

Figure S7. Effect of FDCA concentration on the production of THFDCA over Ru/Al_2O_3.

Conditions:

*1 wt.% FDCA (0.202 g), Cat. Ru/Al_2O_3 (0.1635 g), H_2O 20 mL, H_2 3.1 MPa, T= 50 °C, 4 h.

**5 wt.% FDCA (1.053g), Cat. Ru/Al_2O_3 (0.85 g), H_2O 20 mL, H_2 3.1 MPa, T= 50 °C, 8 h.

***10 wt.% FDCA (2.222 g), Cat. Ru/Al_2O_3 (1.79 g), H_2O 20 mL, H_2 3.1 MPa, T= 50 °C, 20 h.

Effect of Ruthenium loading on the formation of THFDCA

The amount Ru loadings were also varied in order to be optimized.

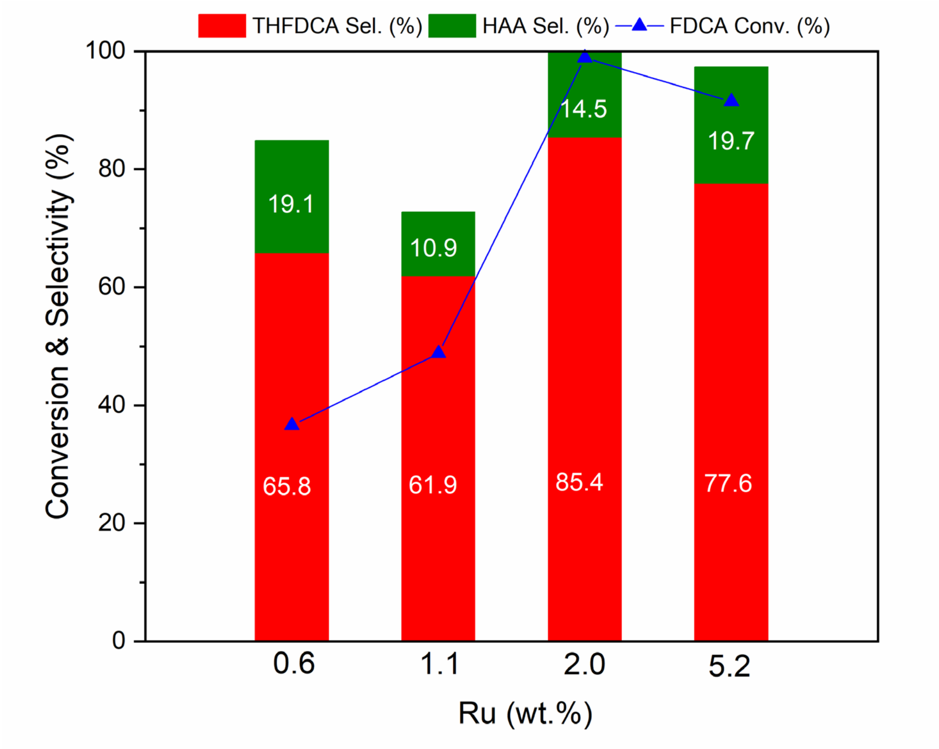

Figure S8. Effect of Ruthenium loading on the formation of THFDCA.

Effect of calcination temperature on AlOOH support and its effect on the conversion of FDCA

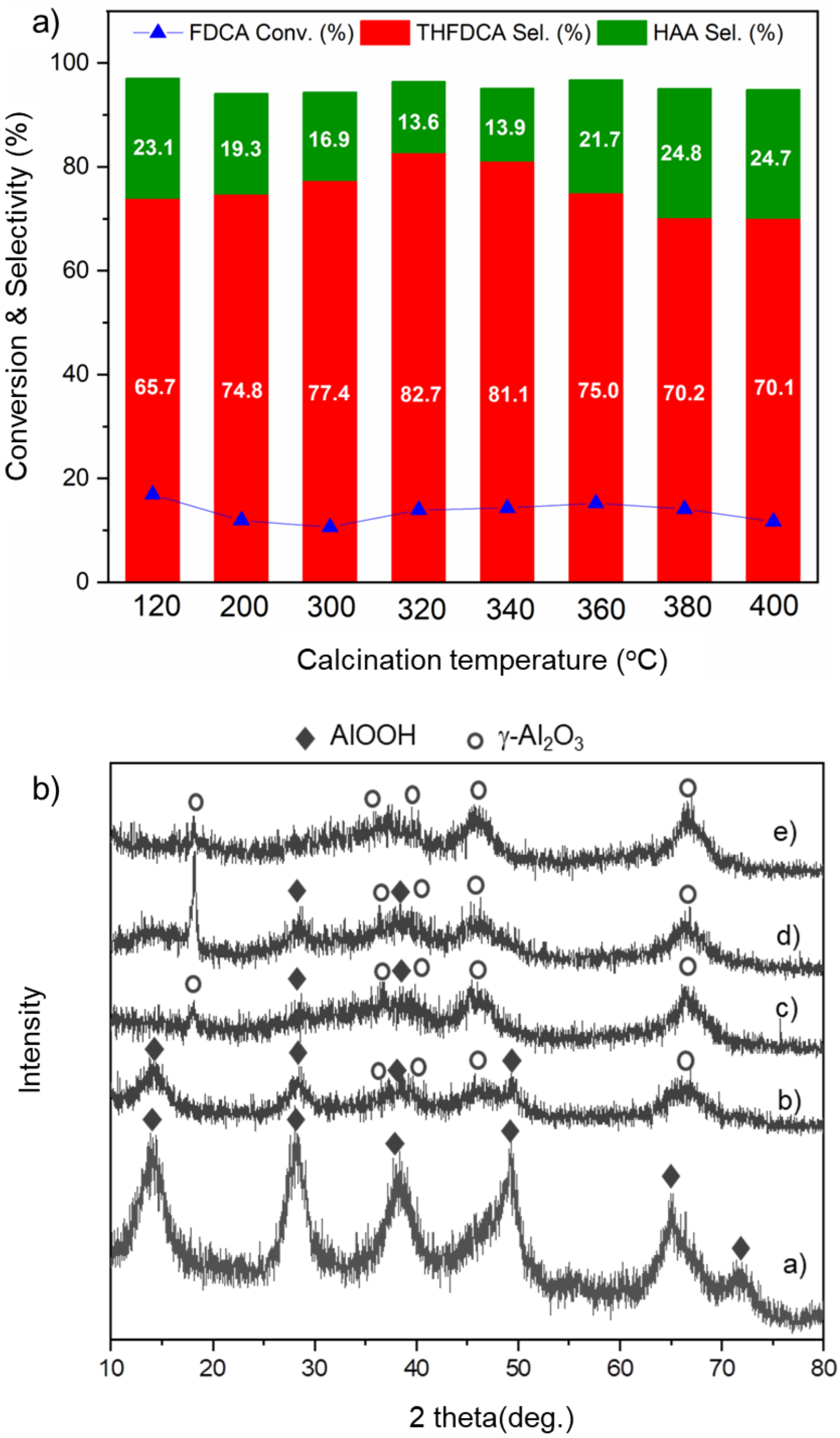

Figure S9. a) Effect of calcination temperatures on the selectivity of Ru/AlOOH; b) XRD spectra of Ru/AlOOH at different calcination temperatures of a) 300 °C, b) 320 °C, c) 340 °C, d) 360 °C, e) 380 °C. Conditions: 1.0 wt.% FDCA (0.202 g), Cat. Ru/support (0.1635 g), Solvent H_2O = 20 mL, P (H_2) = 3.1 MPa, t = 4 h, T = 30 °C.

Section 3: Conversion of THFDCA to AA

Effect of reaction parameters on the formation of AA

As depicted in Figure S10, at the initial stage of the hydrogenolysis (1 h), THFDCA was quickly converted to AA with a 69.7% yield of AA and 15.9% yield of HAA. At a reaction time of 2 h, THFDCA was fully converted into AA with a yield of 98.3%. After complete hydrogenolysis, a longer reaction time (i.e., 3-4 h) did not significantly change the product distributor; AA remained as an almost constant yield. From these results, 2 h was the optimal time for the hydrogenolysis of THFDCA in this reaction system.

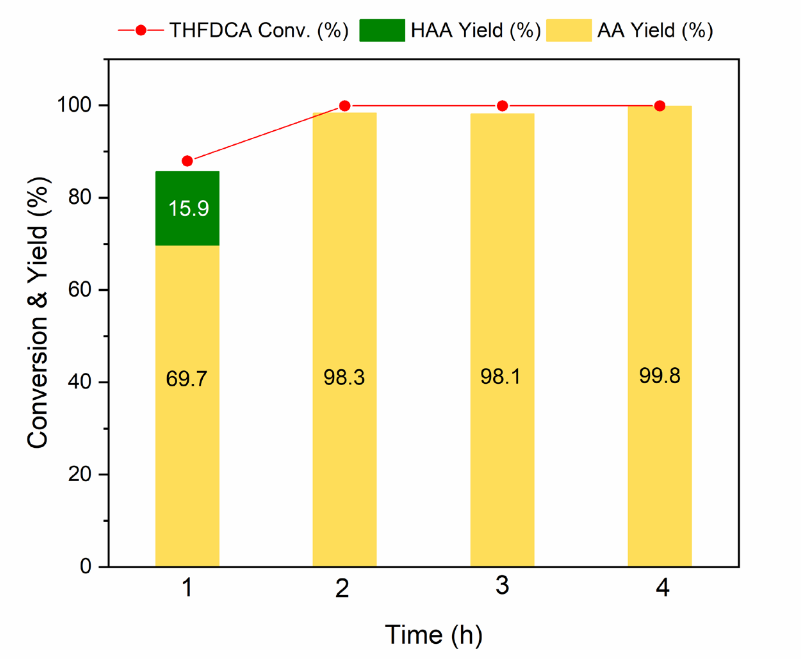

Figure S10. Effect of reaction time on the formation of AA over [MIM$(CH_2)_4SO_3H$]I.

Conditions: THFDCA (0.165 g, 93%), Cat.[MIM$(CH_2)_4SO_3H$]I (1.55 g), H_2 3.4 MPa, T =180 ºC.

As depicted in Figure S11, by increasing temperature from 120 °C to 160 °C, a significant increase in THFDCA conversion (33.2% to 87.2%) and AA yield (16.9% to 66%) was observed, and somewhat increase in the AA selectivity (50.9% to 75.7 %). Further increasing the reaction temperature (160 °C to 180 °C) led to complete conversion of THFDCA (99.9%) as well as an increase in AA yield (66% to 98.3%) and selectivity (75.7% to 98.4%). From these results, 180 °C was chosen as an optimal temperature for the hydrogenolysis of THFDCA.

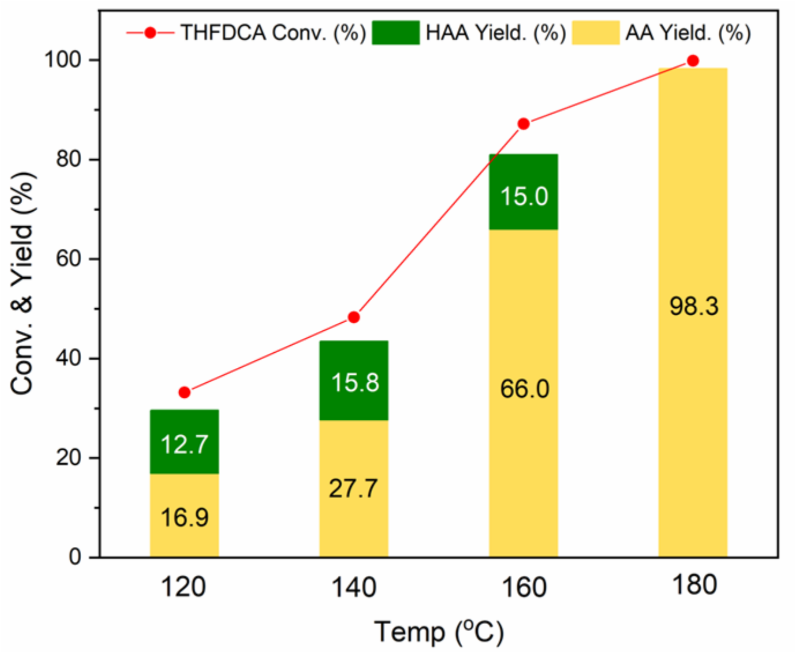

Figure S11. Effect of reaction temperature on the formation of AA over [MIM(CH_2)$_4$$SO_3$H]I.

Conditions: THFDCA (0.165 g, 93%), Cat.[MIM(CH_2)$_4$$SO_3$H]I (1.55 g), H_2 3.4 MPa, t = 2 h.

AA isolation process and regeneration of spent catalyst (IL) after the reaction

The isolation process of AA after the reaction is presented in Scheme S2. The isolated AA was obtained with an 83% yield (Table S4), and its identity was confirmed by using ^{1}H-NMR and ^{13}C-NMR analysis.

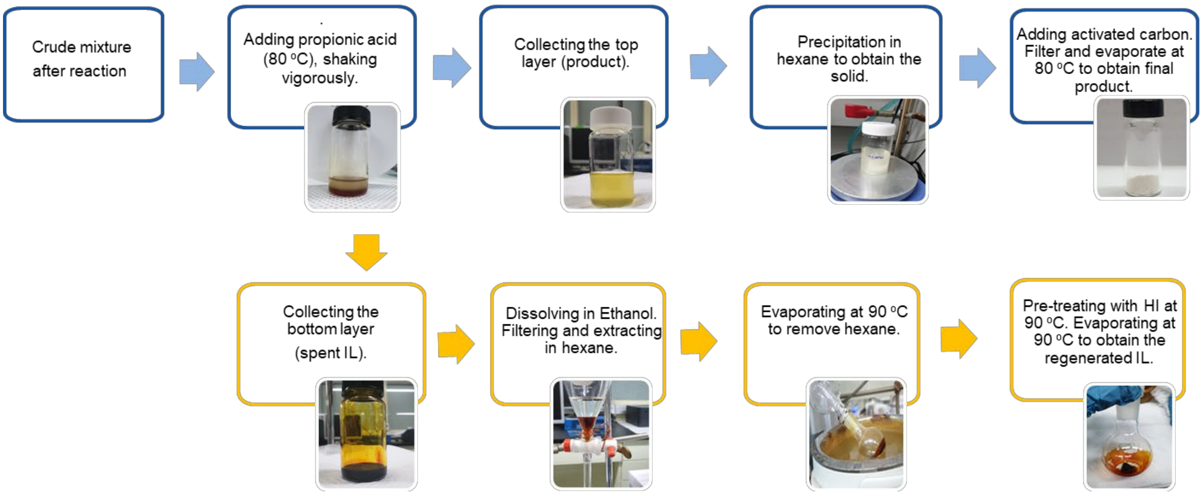

Scheme S1. Isolation of product (AA) (blue box) and regeneration of spent catalyst (IL) (yellow box) after the reaction. Conditions: THFDCA (0.165 g, 0.958 mmol); Cat. [[MIM$(CH_2)_4SO_3H$]I] (1.55 g), t = 2 h, P = 3.4 Mpa, T = 180 °C.

Table S3. Isolation yield and purity of the collected adipic acid (AA) after the reaction.

**Entry**	**AA isolated yield (%)**	**AA purity (%) qNMR analysis**	
1	81.4	98.2	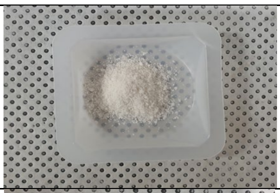
2	88.3	98.7	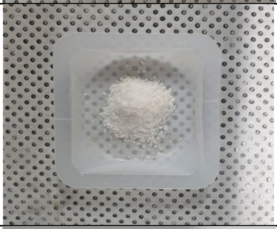
3	80.1	99.6	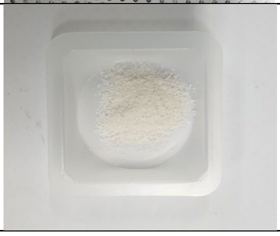
Average	83.3%	98.8%	

The isolated AA purity was analyzed using qNMR with absolute concentration determination. Maleic acid (Sigma-Aldrich, purity ≥99%) was used as an internal standard.

About 5 mg of standard and 5 mg of each isolated AA were added to the NMR tube. Then the mixture was fully dissolved in 0.6 mL of $MeOD_4$ to obtain a solution with a concentration of about 15 mg/mL. ^{1}H NMR spectra of the samples were obtained on Bruker NMR 300 MHz. The spectra of samples were integrated for calculating the purity of the isolated AA (Figure S12, Figure S13, and Figure S14).

The purity of AA was determined using the following formula [2]:

$$P_{AA} = \frac{I_{AA}}{I_{std}} \times \frac{N_{std}}{N_{AA}} \times \frac{M_{AA}}{M_{std}} \times \frac{W_{std}}{W_{AA}} \times P_{std}$$

Where,

P_{AA} : The purity of the isolated AA.

P_{std} : The purity of the standard (Maleic acid, purity≥99%).

I_{AA} : The integrated area of AA signal.

I_{std} : The integrated area of maleic acid.

N_{AA} : The number of AA protons (Sum of -CH_2 proton signal).

N_{std} : The number of maleic acid protons (Sum of =CH proton signal).

M_{AA} : The molecular mass of AA (g/mol).

M_{std} : The molecular mass of maleic acid (g/mol).

W_{AA}: The gravimetric weight of AA (mg).

W_{std} : The gravimetric weight of maleic acid (mg).

Table S4. Purity evaluation information of the isolated AA based on H^1 qNMR spectra.

Entry	Compound	Integrals	Purity Evaluation
1 (Figure S12)	(+) Isolated AA: M_{AA} = 146.14 g/mol, W_{AA} = 6.2 mg. (+) Standard: M_{std} = 116.07 g/mol, W_{std} = 5.9 mg, P_{std} = 99%	(+) Isolated AA: I_{AA} = 6.66 N_{AA} = 8 (Sum of -CH_2 proton signals). (+) Standard: I_{std} = 2, N_{std} = 2 (Sum of =CH proton signals).	P_{AA} = 98.7%
2 (Figure S13)	(+) Isolated AA: M_{AA} = 146.14 g/mol, W_{AA} = 4.8 mg. (+) Standard: M_{std} = 116.07 g/mol, W_{std} = 4.7 mg, P_{std} = 99%	(+)Isolated AA: I_{AA} = 6.44 N_{AA} = 8 (Sum of -CH_2 proton signals). (+) Standard: I_{std} = 2, N_{std} = 2 (Sum of =CH proton signals).	P_{AA} = 98.2%
3 (Figure S14)	(+) Isolated AA: M_{AA} = 146.14 g/mol, W_{AA} = 4.8 mg. (+) Standard: M_{std} = 116.07 g/mol, W_{std} = 5.8 mg, P_{std} = 99%	(+)Isolated AA: I_{AA} = 5.29 N_{AA} = 8 (Sum of -CH_2 proton signals). (+) Standard: I_{std} = 2, N_{std} = 2 (Sum of =CH proton signals).	P_{AA} = 99.6%

Section 4: Two-step pathway for producing AA from a high concentration of FDCA.

The production of AA was conducted via a two-step pathway from the hydrogenation of FDCA and subsequent ring-opening of THFDCA.

The hydrogenation of 10 wt.% FDCA was performed over Ru/Al_2O_3 in a high-pressure reactor under the conditions of 50 °C, 450 psi for 48 h. The mixture product of THFDCA and HAA was isolated and purified before applying to the ring-opening reaction for producing AA.

Table S5. Two-step pathway for producing AA from FDCA

Entry	Catalyst	Substrate	FDCA Conversion (%)	THFDCA Yield (%)	HAA Yield (%)
1	Ru/Al_2O_3	10 wt.% FDCA	98	78.7	19

Conditions: 10 wt.% FDCA (3.4 g), 30 mL H_2O, MR sub/Ru=20, Ru/Al_2O_3 = 2.75 g , 50 °C, 3.1 MPa, 48 h.

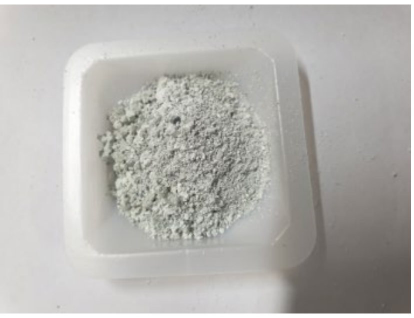

The mixture of THFDCA + HAA was isolated after the reaction.

Entry	Catalyst	Substrate	THFDCA +HAA Conversion (%)	AA Yield (%)
2	$[MIM(CH_2)_4SO_3H]I$	Isolated THFDCA + HAA	>99.9	97.3

Conditions: THFDCA + HAA (2.4 g), IL (2.01 g),non-solvent, MR sub/Cat =0.22, 180 °C, 3.4 MPa, 4 h.

Section 5: ^{1}H-NMR and ^{13}C-NMR spectra

^{1}H-NMR spectra of the isolated product AA.

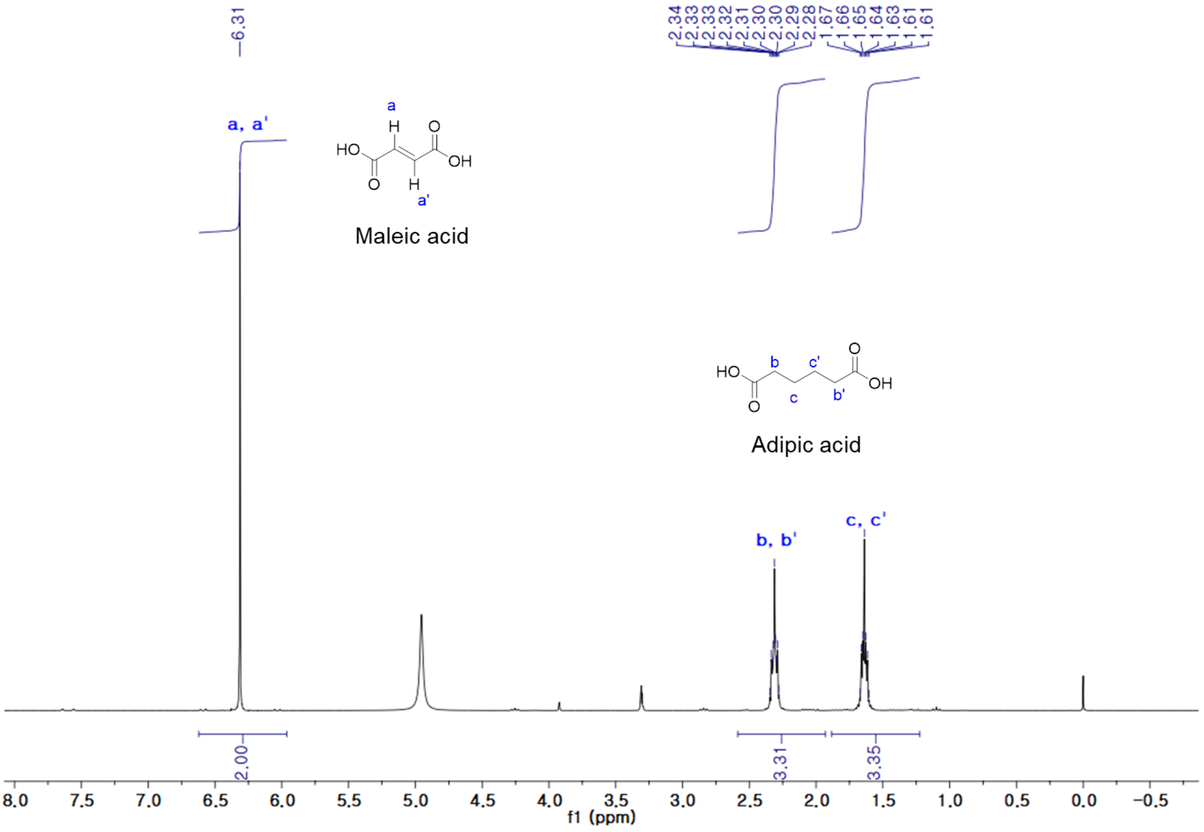

Figure S12. Integrated peaks of ^{1}H qNMR spectra of the first isolated AA using maleic acid as an internal standard in $MeOD_4$ (Entry 1). ^{1}H qNMR (300 MHz, $MeOD_4$): adipic acid δ 2.31 (m, 4H); 1.66 (m, 4H); maleic acid δ 6.31 (s, 2H).

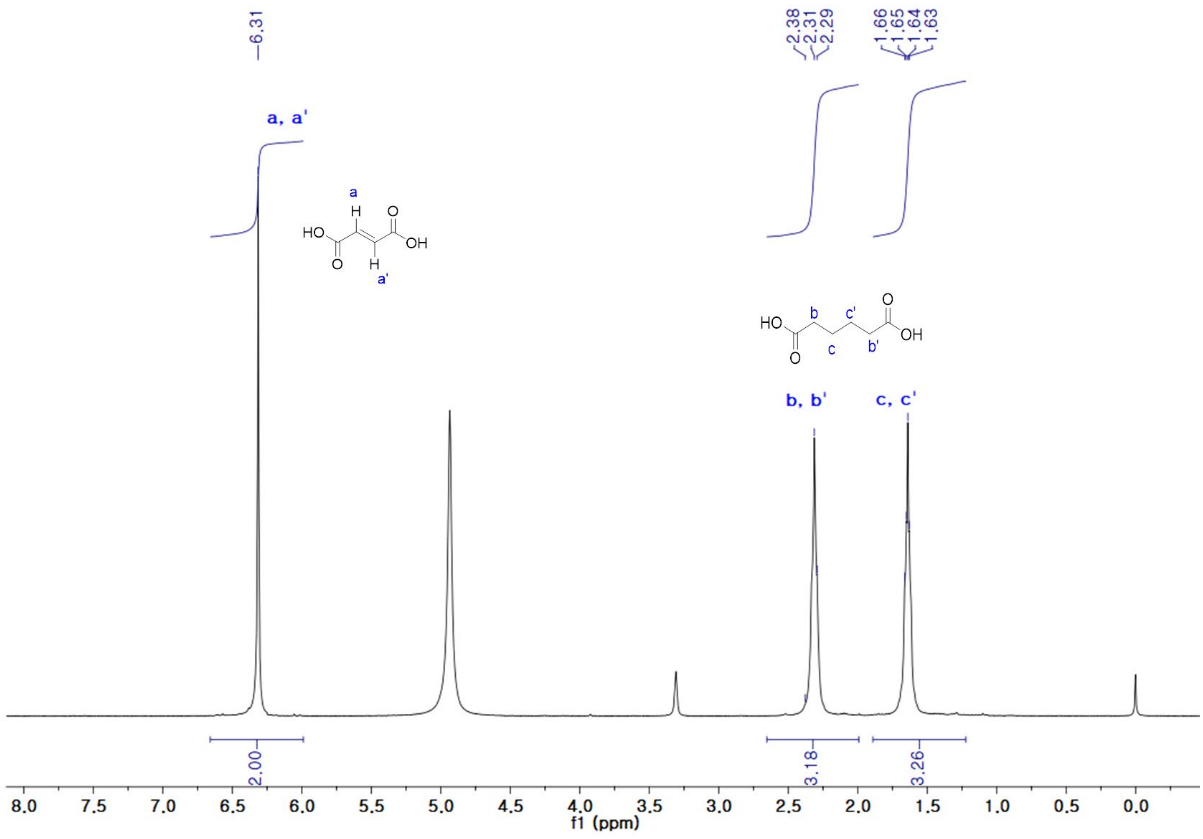

Figure S13. Integrated peaks of ^{1}H qNMR spectra of the second isolated AA using maleic acid as an internal standard in $MeOD_4$ (Entry 2). ^{1}H qNMR (300 MHz, $MeOD_4$): adipic acid δ 2.33 (m, 4H); 1.64 (m, 4H); maleic acid δ 6.31 (s, 2H).

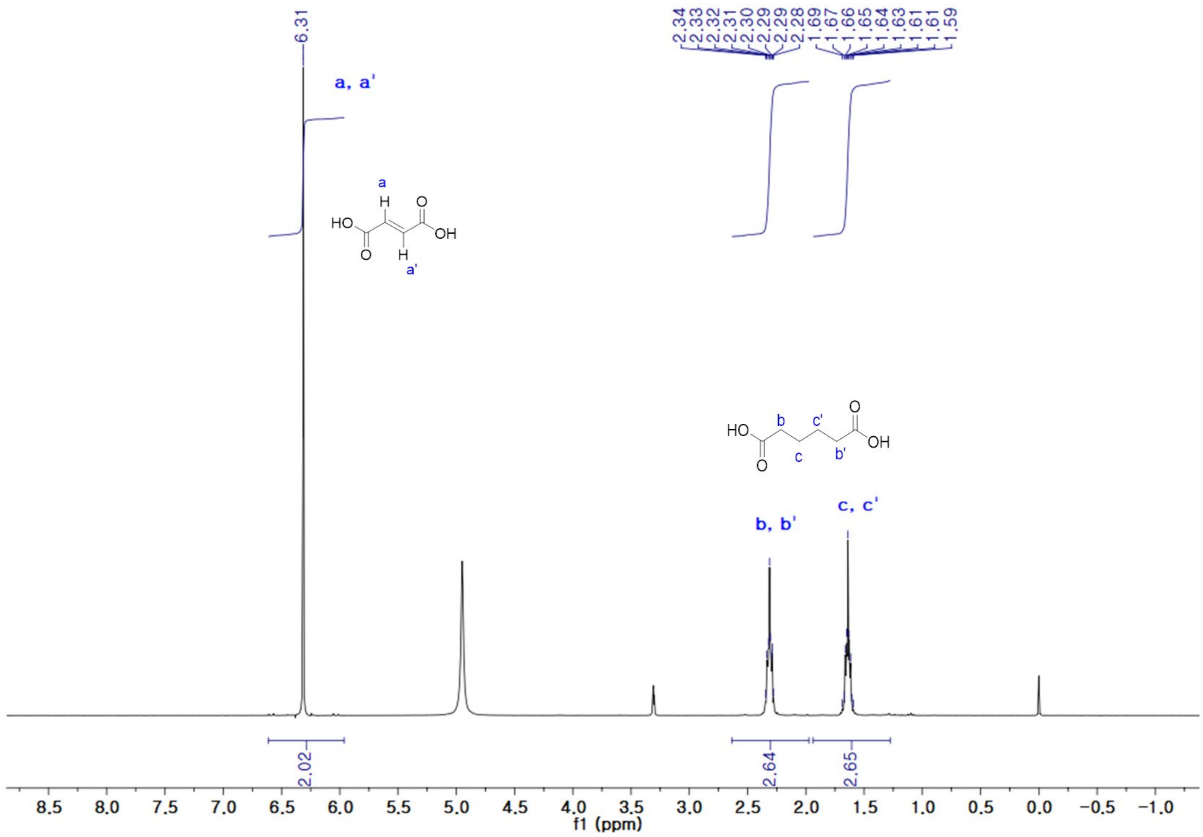

Figure S14. Integrated peaks of ^{1}H qNMR spectra of the third isolated AA using maleic acid as an internal standard in $MeOD_4$ (Entry 3). ^{1}H qNMR (300 MHz, $MeOD_4$): adipic acid δ 2.31 (m, 4H); 1.64 (m, 4H); maleic acid δ 6.31 (s, 2H).

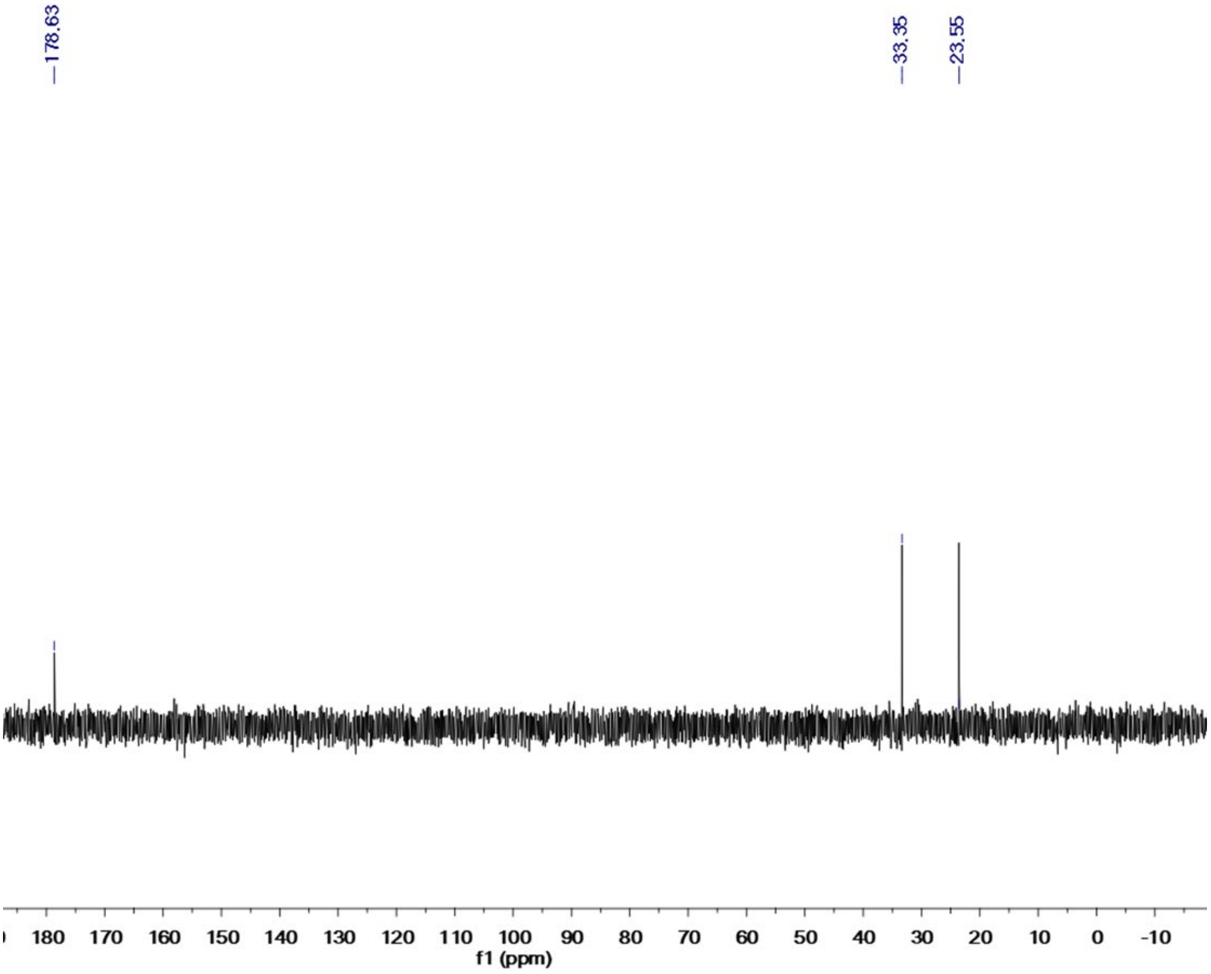

Figure S15. ^{13}C-NMR spectra of the isolated AA. ^{13}C-NMR (300 MHz, D_2O): δ 178.6, 33.3, 23.5.

^{1}H-NMR spectra of the $MIM(CH_2)_4SO_3$

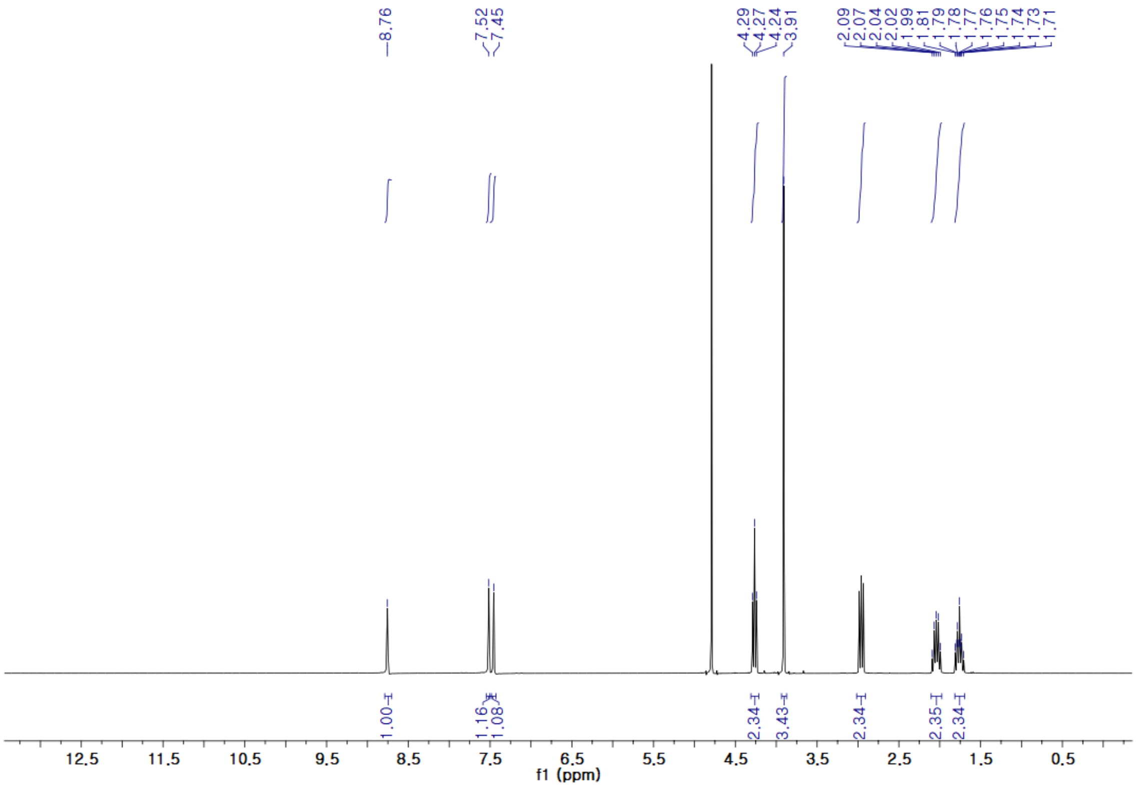

Figure S16. ^{1}H-NMR spectra of [MIM-BS]. ^{1}H-NMR (300 MHz, D_2O): δ (ppm): 1.76 (m, 2H), 2.02 (m, 2H), 2.96 (m, 2H), 3.91 (s, 3H), 4.27 (t, 2H), 7.45 (s, 1H), 7.52 (s, 1H), 8.76 (s, 1H). The obtained spectroscopic data were in agreement with the reported data for this compound [3].

^{1}H-NMR spectra of the fresh [MIM(CH$_2$)$_4$SO$_3$H]I and the spent [MIM(CH$_2$)$_4$SO$_3$H]I after 4th cycles.

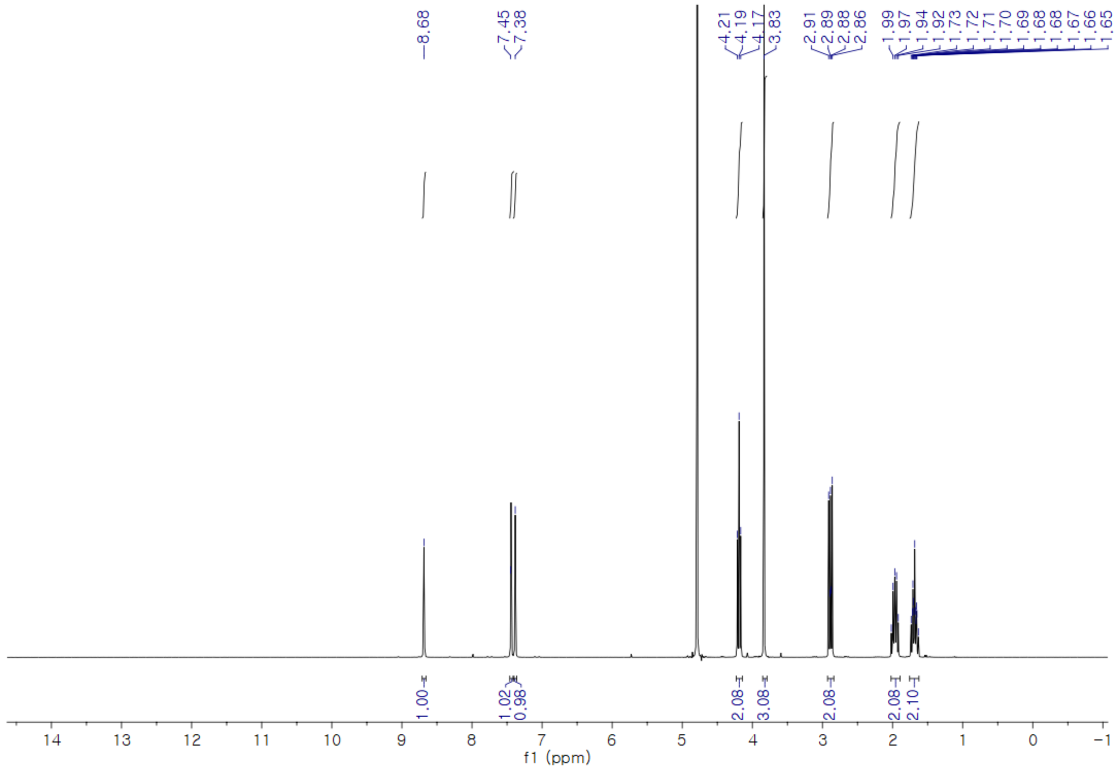

Figure S17. ^{1}H-NMR spectra of fresh [MIM(CH$_2$)$_4$SO$_3$H]I. ^{1}H-NMR (300 MHz, D_2O): δ (ppm) 8.68 (s, 1H), 7.45 (s, 1H), 7.36 (s, 1H), 4.19 (t, 2H), 3.83 (s. 3H), 2.89 (m, 2H), 1.97 (m, 2H), 1.68 (m, 2H).

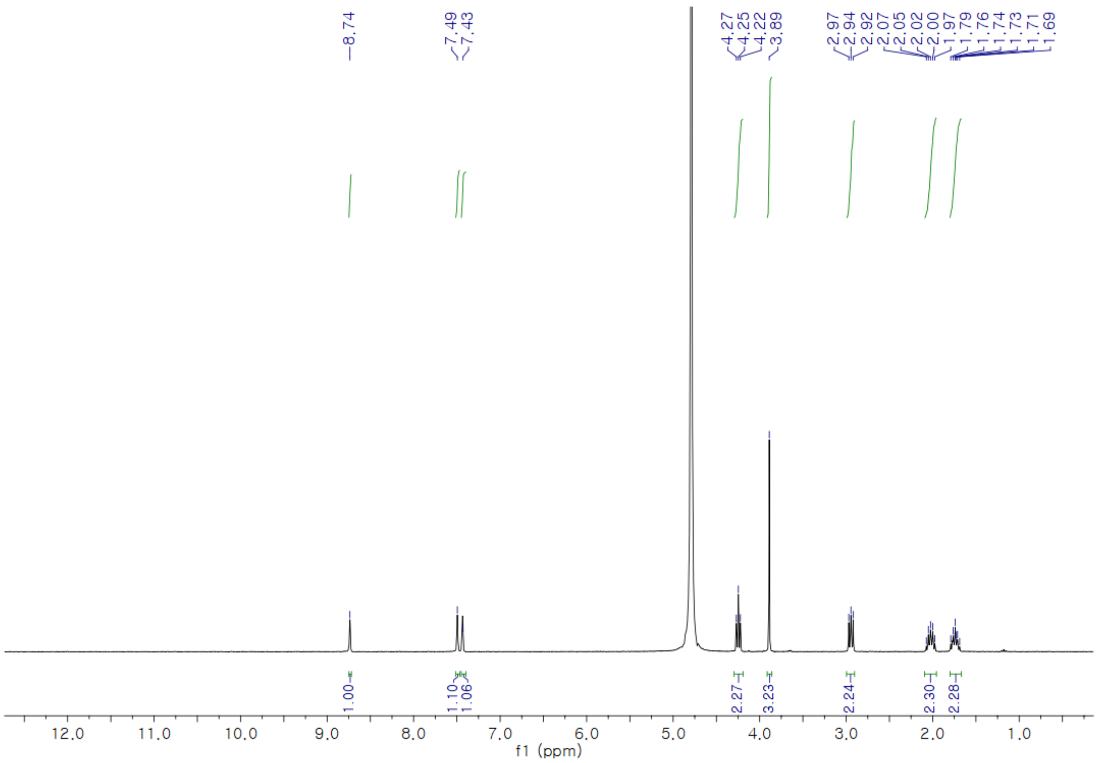

Figure S18. ^1H-NMR spectra of 4th reused $[MIM(CH_2)_4SO_3H]I$. ^1H-NMR (300 MHz, D_2O): δ (ppm) 8.74 (s, 1H), 7.49 (s, 1H), 7.43 (s, 1H), 4.25 (t, 2H), 3.89 (s, 3H), 2.94 (m, 2H), 2.02 (m, 2H), 1.74 (m, 2H).

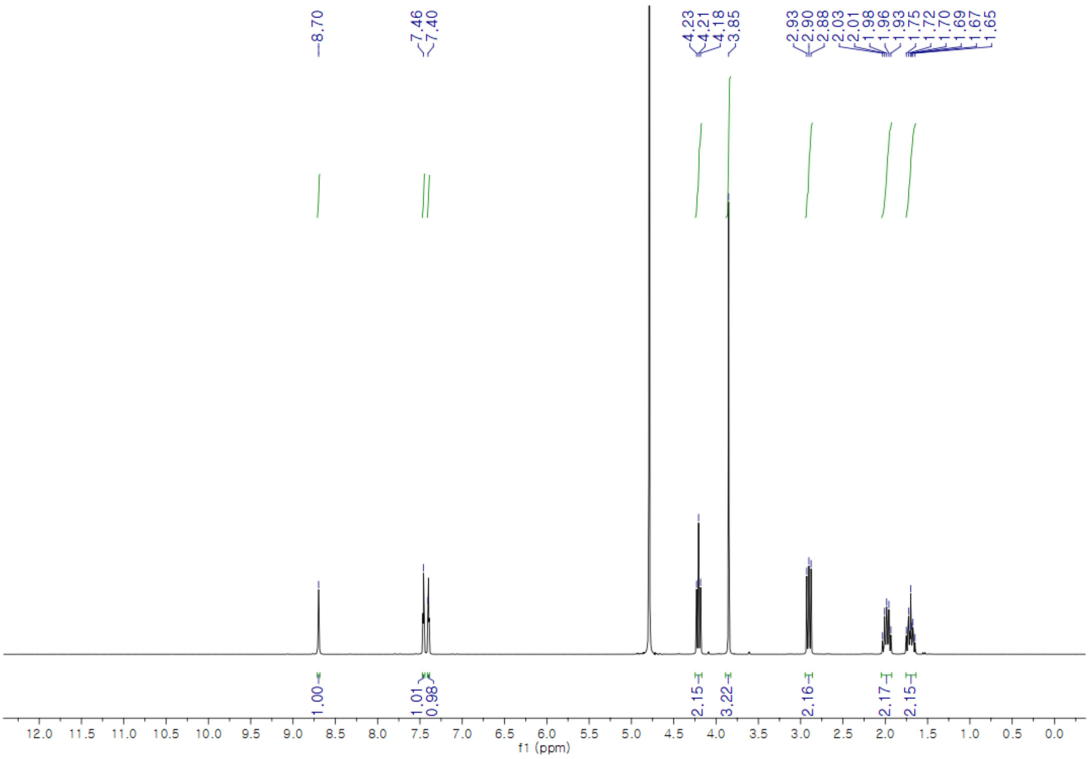

Figure S19. ^{1}H-NMR spectra of [MIM(CH$_2$)$_4$SO$_3$H]Br. ^{1}H-NMR (300 MHz, D_2O): δ (ppm) = 8.7 (s, 1H), 7.46 (s, 1H), 7.40 (s, 1H), 4.21 (t, 2H), 3.85 (s, 3H), 2.9 (m, 2H), 2.01 (m, 2H), 1.7 (m, 2H).

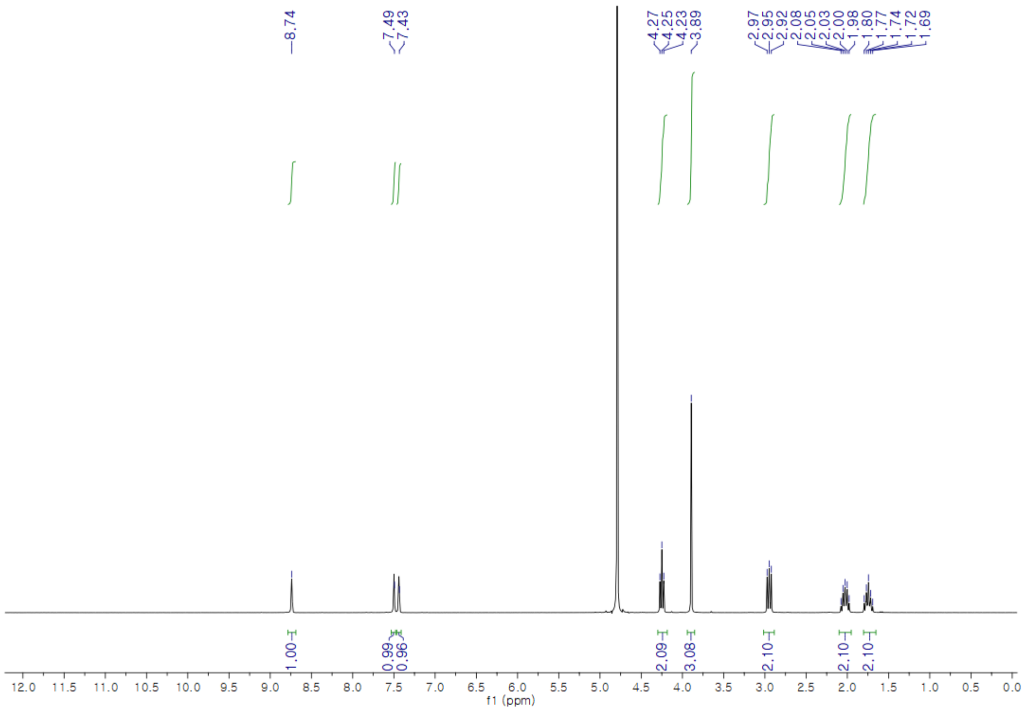

Figure S20. ^{1}H-NMR spectra of [MIM(CH$_2$)$_4$SO$_3$H]Cl. ^{1}H-NMR (300 MHz, D_2O): δ (ppm) = 8.74 (s, 1H), 7.49 (s, 1H), 7.43 (s, 1H), 4.25 (t, 2H), 3.89 (s, 3H), 2.95 (m, 2H), 2.03 (m, 2H), 1.74 (m, 2H).

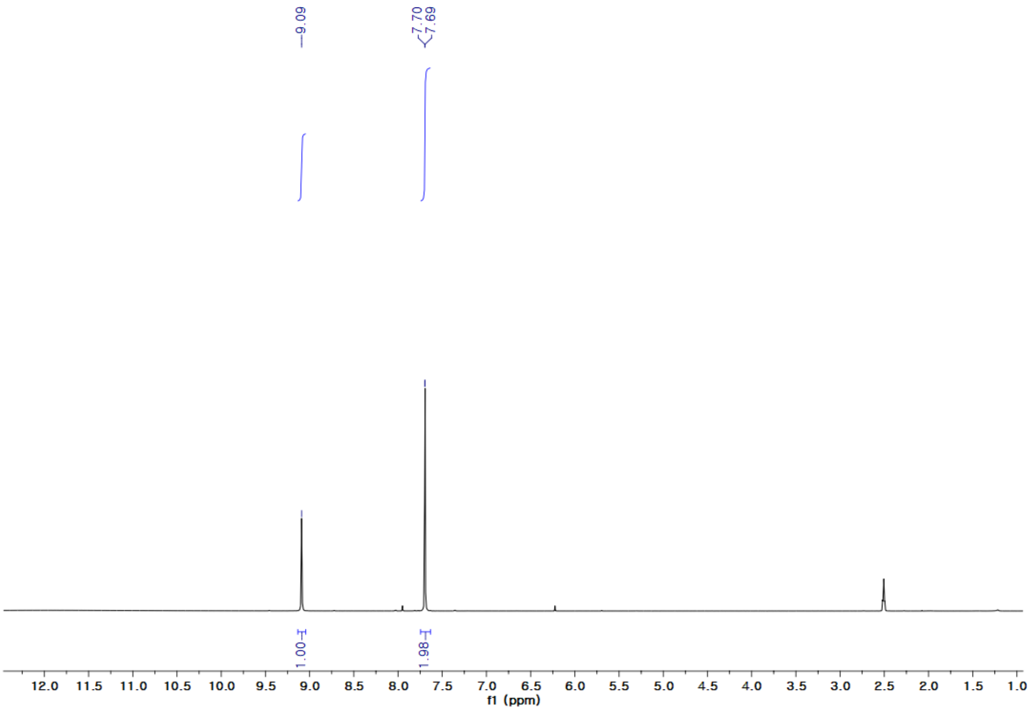

Figure S21. ^{1}H-NMR spectra of [IM]I. ^{1}H-NMR (300 MHz, DMSO): δ (ppm) 9.09 (s, 1H), 7.70 (s, 1H), 7.69 (s, 1H). The obtained spectroscopic data were in agreement with the reported data for this compound [4].

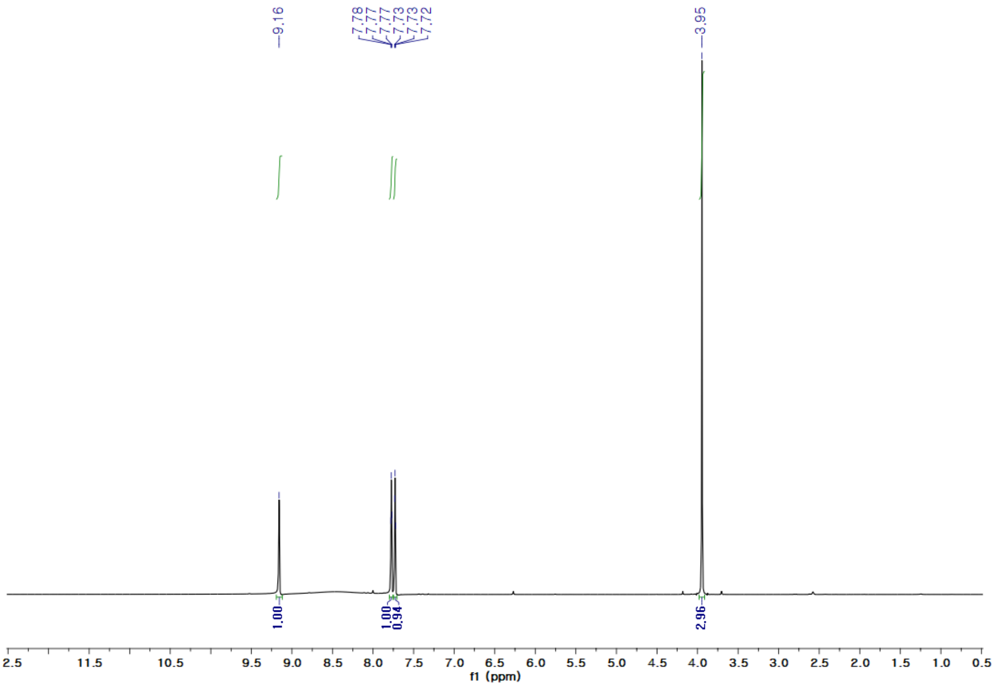

Figure S22. ^{1}H-NMR spectra of [MIM]I. ^{1}H-NMR (300 MHz, DMSO): δ (ppm) 9.16 (s, 1H), 7.77 (t, 1H), 7.73 (t, 1H), 3.95 (s, 3H). The obtained spectroscopic data were in agreement with the reported data for this compound [4].

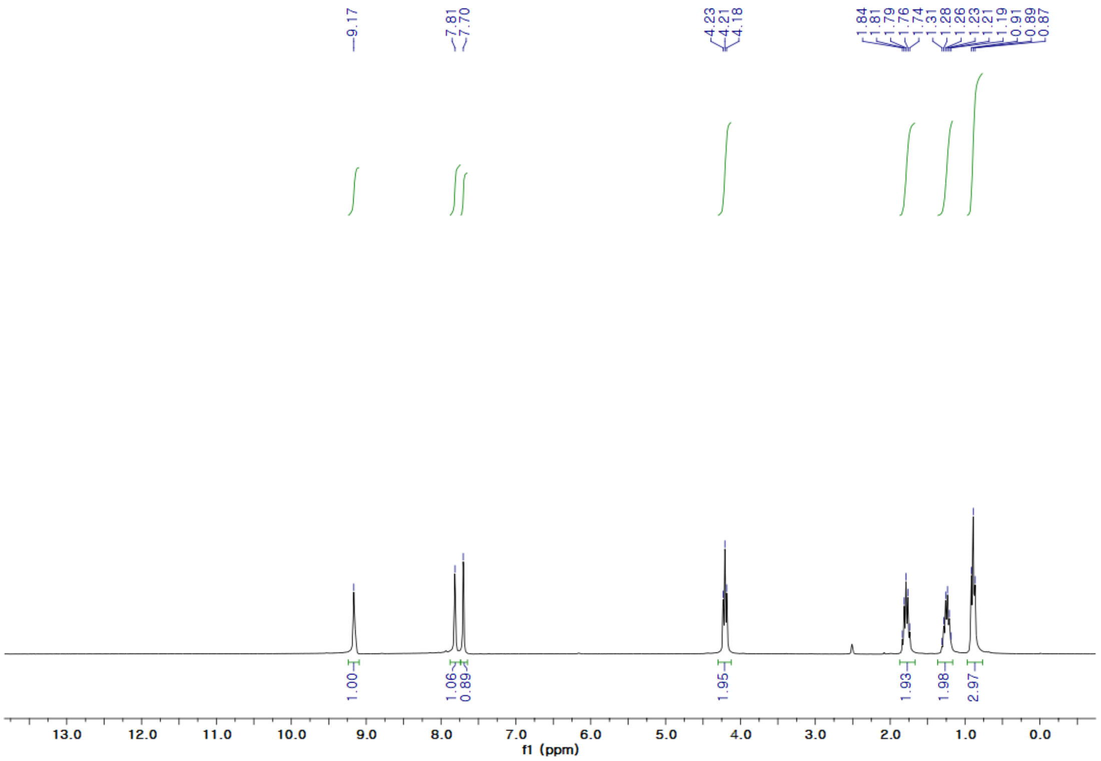

Figure S23. ^{1}H-NMR spectra of [BIM]I. ^{1}H-NMR (300 MHz, DMSO): δ (ppm) 9.17 (s, 1H), 7.81 (s, 1H), 7.7 (s, 1H), 4.21 (t, 2H), 1.79 (m, 2H), 1.23 (m, 2H), 0.89 (t, 3H), 1.23 (s, 2H), 1.79 (s, 2H), 4.21 (s, 2H), 7.70 (s, 1 H), 7.81 (s, 1H), 9.17 (s, 1 H). The obtained spectroscopic data were in agreement with the reported data for this compound [4].

Section 6:

References

[1] F. Wang, C. Li, X. Zhang, M. Wei, D. G. Evans, X. Duan, *J. Catal.* **2015**, *329*, 177-186.
[2] T. Schoenberger, *Anal. Bioanal. Chem.* **2012**, *403*, 247-254.
[3] S. Liu, S. Tan, B. Bian, H. Yu, Q. Wu, Z. Liu, F. Yu, L. Li, S. Yu, X. Song, Z. Song, *RSC Adv.* **2018**, *8*, 19551-19559.
[4] S. G. Kalghatgi, B. M. Bhanage, *J. Mol. Liq.* **2019**, *281*, 70-80.
